# Influence of Enriched Environment on Viral Encephalitis Outcomes: Behavioral and Neuropathological Changes in Albino Swiss Mice

**DOI:** 10.1371/journal.pone.0015597

**Published:** 2011-01-11

**Authors:** Aline Andrade de Sousa, Renata Reis, João Bento-Torres, Nonata Trévia, Nara Alves de Almeida Lins, Aline Passos, Zaire Santos, José Antonio Picanço Diniz, Pedro Fernando da Costa Vasconcelos, Colm Cunningham, Victor Hugh Perry, Cristovam Wanderley Picanço Diniz

**Affiliations:** 1 Universidade Federal do Pará (UFPA), Instituto de Ciências Biológicas, Laboratório de Investigações em Neurodegeneração e Infecção, Hospital Universitário João de Barros Barreto, Belém, Brazil; 2 Instituto Evandro Chagas (IEC), Departamento de Arbovirologia e Febres Hemorrágicas, Ananindeua, Brazil; 3 Departamento de Patologia, Universidade do Estado do Pará, Belém, Brazil; 4 School of Biochemistry and Immunology, Trinity College Institute of Neuroscience, Trinity College, Dublin, Ireland; 5 School of Biological Sciences, University of Southampton, Southampton, United Kingdom; University of Nebraska Medical Center, United States of America

## Abstract

An enriched environment has previously been described as enhancing natural killer cell activity of recognizing and killing virally infected cells. However, the effects of environmental enrichment on behavioral changes in relation to virus clearance and the neuropathology of encephalitis have not been studied in detail. We tested the hypothesis that environmental enrichment leads to less CNS neuroinvasion and/or more rapid viral clearance in association with T cells without neuronal damage. Stereology-based estimates of activated microglia perineuronal nets and neurons in CA3 were correlated with behavioral changes in the Piry rhabdovirus model of encephalitis in the albino Swiss mouse. Two-month-old female mice maintained in impoverished (IE) or enriched environments (EE) for 3 months were behaviorally tested. After the tests, an equal volume of Piry virus (IEPy, EEPy)-infected or normal brain homogenates were nasally instilled. Eight days post-instillation (dpi), when behavioral changes became apparent, brains were fixed and processed to detect viral antigens, activated microglia, perineuronal nets, and T lymphocytes by immuno- or histochemical reactions. At 20 or 40 dpi, the remaining animals were behaviorally tested and processed for the same markers. In IEPy mice, burrowing activity decreased and recovered earlier (8–10 dpi) than open field (20–40 dpi) but remained unaltered in the EEPy group. EEPy mice presented higher T-cell infiltration, less CNS cell infection by the virus and/or faster virus clearance, less microgliosis, and less damage to the extracellular matrix than IEPy. In both EEPy and IEPy animals, CA3 neuronal number remained unaltered. The results suggest that an enriched environment promotes a more effective immune response to clear CNS virus and not at the cost of CNS damage.

## Introduction

Sublethal encephalitis following viral infections is known to affect behavior and the immune response, and viral diseases of the central nervous system (CNS) represent a significant proportion of neurological disabilities, particularly in poor countries [1]. Emerging virus infections of the CNS are mainly associated with RNA viruses and many that cause neurologic disease [2]. The rhabdoviruses are part of the broad group of negative-strand RNA viruses, a group that includes a number of medically relevant viruses such as avian influenza, measles, Ebola, and vesicular stomatitis virus (VSV) [3]. Because VSV has limited human pathogenicity, it has been used as a model of rhabdoviruses in both *in vitro* and *in vivo* studies investigating viral adaptive and host immune responses [4], [5].

An enriched environment (EE) was previously described as enhancing natural killer (NK) cell activity, including their recognition and killing of virally infected cells [6]. An enriched environment has been defined as social interactions with con-specifics and a stimulation of exploratory and motor behavior with a variety of toys, ladders, tunnels, rope, bridges, and running wheels for voluntary physical exercise changed periodically, as opposed to an impoverished environment with reduced to social interactions [7]. Rats reared under EE conditions present immune cell recruitment with a higher number of activated microglia than control rats, and these ramified microglial cells resemble the neuroprotective phenotype of microglia activated by T-cell–derived cytokines [8].

A variety of viruses on the surface of host cells target specialized features of the extracellular matrix, known as perineuronal nets (PNs) [9], which consist of glycosaminoglycans. Virus affinity for glycosaminoglycans is a determinant of cell tropism, loss of invasiveness, and reduced efficiency in viral spreading through the circulation [10], [11], [12]. Because environmental enrichment increases the number of PNs [13], [14], of interest is whether or not viral spreading and neuroinvasion are reduced in animals housed under the EE condition.

Infections of the CNS with cytopathic neurotropic viruses, such as vesiculoviruses, require the parenchymal penetration of dendritic cells, T lymphocytes, and microglial activation for virus clearance and survival [15], [16], [17]. Because T cell migration to the brain parenchyma enhances viral clearance in VSV encephalitis [18], we tested the hypothesis that an EE may increase T cell migration to the parenchyma and promote faster virus clearance from the brain.

Thus, in the present report, we induced Piry viral encephalitis in an adult albino Swiss mouse model housed in an impoverished environment (IE) or EE to investigate the hypothesis that an EE may reduce neuropathological damage and behavioral changes and promote less CNS invasion and/or faster virus clearance from the brain. We found that compared to infected IE animals, infected EE animals presented less viral neuroinvasion, less microglial activation, less damage in the specialized extracellular matrix, greater infiltration of CD3-immunolabeled T-lymphocytes in the brain parenchyma, and reduced behavioral changes.

## Results

### Piry virus encephalitis, cell targets, and areas of neuroinvasion

**Figure 1:**
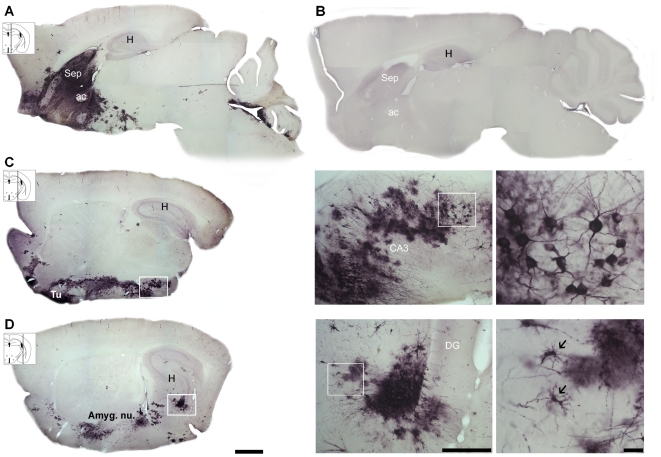
Cellular and neuroanatomical viral targets. Photomicrographs from Piry virus immunostained parasagittal sections of infected (A, C, and D) and uninfected (B) animals to illustrate cellular and targets areas at 8 dpi. Note the dendrites, axon fibers, and cell somata of neurons with altered cell appendages and microglia-like cells (arrows) in infected but not in control mice. Immunostaining includes the olfactory bulb, olfactory nuclei, olfactory tubercles, piriform cortex, septum (Sep), amygdala (Amyg. nu.), ventral hippocampus, hippocampal fimbria (F), polymorphic layer of the ventral dentate gyrus (DG), and CA3. ac: anterior commissure; H: hippocampus. Scale bars: low power: 1.0 mm; medium power: 250 µm; high power: 25 µm.

Figure 1 illustrates the cellular targets and areas of neuroinvasion in adult female mice at 8 days post-instillation (dpi). Piry viral antigens in the brain parenchyma were revealed in the cytoplasm of infected cells that stained positive for virus proteins. This feature is consistent with the fact that viral proteins associated with RNA viruses are located in the cytoplasm. Immunolabeled dendrites, axon fibers, and cell somata showing small dots of dense viral antigen accumulation were detected in the parenchyma, mainly in the olfactory pathways, including the olfactory bulb (not illustrated), olfactory nuclei, olfactory tubercles, piriform cortex, and amygdala as well as the septum, ventral hippocampus, hippocampal fimbria, and polymorphic layer of the ventral dentate gyrus. Viral antigens were detected in both axons and dendrites, which frequently presented many abnormal varicosities sometimes associated with closely adjacent immunolabeled glial cells (not illustrated), suggesting a possible interaction between diseased neurons and glia. Because our previous work had revealed that Piry virus neuroinvasion targets a variety of brain areas including hippocampal CA3 fields (unpublished data) inducing apoptosis and picknosis [19] in that region, we decided to estimate the number of activated microglia, perineuronal nets and neurons of CA3. Pyramidal CA3 neurons and non-pyramidal stellate neurons of the polymorphic layer of the dentate gyrus and glial cells were invaded equally, and a diffuse pattern of immunostaining in the extracellular space was frequently found (Fig. 1).

### Response to Piry virus

**Figure 2:**
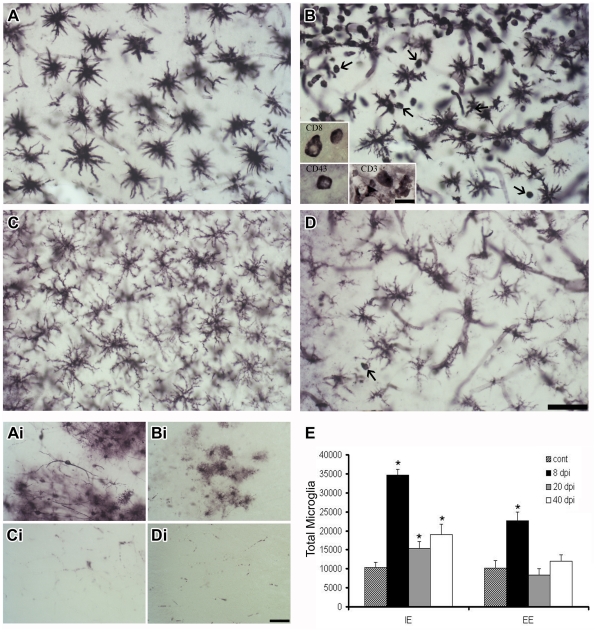
Viral neuroinvasion and microglial inflammatory response. Photomicrographs from IEPy (A, C, Ai, and Ci) and EEPy (B, D, Bi, and Di) parasagittal sections to illustrate the intensity of microglial activation and the presence of Piry virus antigens in the brain parenchyma. Note that the ventral CA3 (Ai, Bi), but not dorsal (Ci, Di), is intensely immunostained for Piry virus antigens and that morphologically activated microglia are more prominent in the ventral CA3 (A, B) as compared to the dorsal CA3 (C, D). Tomato lectin-positive small rounded cells other than activated microglia are indicated in EEPy (arrows). High-power pictures of immunolabeled CD-3–, CD-8–, and CD43-positive cells from the same region are illustrated in the insert (B). E: Total number and respective standar error bars (s.e.m.) of microglia estimations in CA3 at 8, 20, and 40 dpi and in control animals. Note the higher number of microglias in IEPy and that the numbers of activated microglia remained above control values, even after 40 dpi in IEPy, whereas EEPy returned to control levels at 20 dpi. Two-way ANOVA for environmental conditions (F = 20.71; *p*<0.0001) and survival time (F = 38.73; *p*<0.0001) with a significant interaction between these variables (F = 4.18; *p* = 0.0138). IEcont, EEcont: impoverished and enriched environment control groups, IEPy, EEPy: impoverished and enriched environment infected groups. Scale bars: 25 µm (low power) and 10 µm (high power).

Tomato lectin binds several major lymphocyte and microglial cell surface glycoproteins and is readily detected by a simple two-step reaction, revealing the distribution of T cells and both quiescent and activated microglia [20], [21]. Widespread microglial activation with an altered morphological phenotype was found at 8 dpi in the same areas where viral antigens were detected in both IE mice infected with Piry (IEPy) (Fig. 2Ai and Ci) and infected EE mice (EEPy) (Fig. 2Bi and Di), with a greater intensity in IE (Fig. 2A and C) than EE (Fig. 2B and D) animals. We have also found less viral immunolabeling in the brain sections from EEPy animals compared to IEPy (Fig. S1). In both IEPy and EEPy mice, at this time point, dorsal hippocampus showed less neuroinvasion (Fig. 2Ci and Di) than ventral hippocampus (Fig. 2Ai and Bi). Small rounded tomato lectin-positive cells that differed from the phagocytic phenotype of microglia were also detected around 8 dpi and were particularly prominent in the EEPy group (arrows in Fig. 2B and D). In the EEPy hippocampus, small cells were immunostained by anti-CD3, anti-CD8, and anti-CD43 (insert in Fig. 2B), suggesting that the EE had increased the occurrence of infiltrating T cells in the infected areas. After 20 dpi or later when the Piry virus had already been cleared from the brain parenchyma, infiltrated tomato lectin-positive cells was virtually absent. Uninfected EE control mice were devoid of rounded tomato lectin-positive cells and CD3 immunolabeled cells in the brain parenchyma.

Figure 2E represents average values of optical fractionator estimations of CA3-activated microglial cells in each environmental condition at 8, 20, and 40 dpi after histochemical staining with *Lycopersicum esculentum* aglutinin. Microglial estimations revealed a significant increase at 8 dpi in both IEPy and EEPy groups compared to the respective controls, but EEPy animals recovered to control values faster than did IEPy mice. Despite a significant reduction in microglia numbers in IEPy animals by 20 and 40 dpi from peak numbers at 8 dpi, total numbers remained above control levels, whereas the number of microglia in the EEPy group was indistinguishable from respective control animals at 20 and 40 dpi.

**Figure 3:**
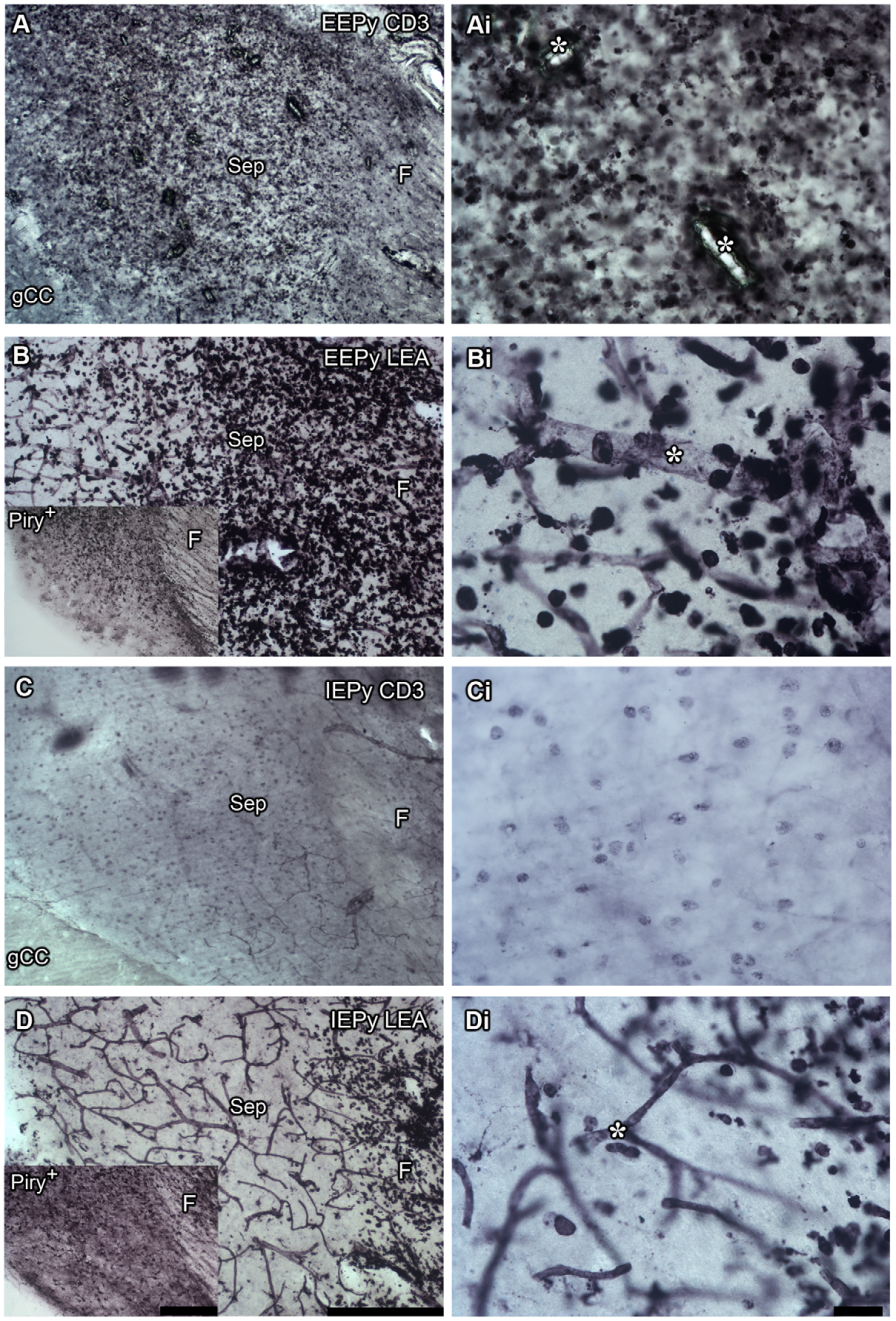
Inflammatory cell infiltration into the brain parenchyma. Photomicrographs from anti-CD3 (A and C) and tomato lectin labeling (B and D) and Piry virus immunolabeling (insert) from EEPy (A and B) and IEPy (C and D) from fimbria-septum parasagittal sections at 8 dpi. High-power pictures from the left column are depicted on the right column, side by side. There is a higher intensity of anti-CD3 immunolabeling and the presence of a higher number of rounded tomato lectin-positive cells in EEPy as compared with IEPy. In EEPy, the higher level of CD3 immunostaining is visible in the septum whereas tomato lectin histochemical staining seems to be more intense in the hippocampal fimbria. The higher amount of CD3 or tomato-lectin rounded stained cells is correlated with a lesser intensity of Piry virus immunolabeling. Sep: septum; F: fimbria; gcc: genus of corpus callosum. (*) indicates blood vessels. IEcont, EEcont: impoverished and enriched environment control groups, IEPy, EEPy: impoverished and enriched environment infected groups. Scale bars: 25 µm (low power) and 10 µm (high power).

Figure 3 illustrates the presence of virus antigens and inflammatory cells in the septum and hippocampal fimbria. It shows photomicrographs from anti-CD3 (A and C) and Piry virus (insert) immunolabeling and tomato lectin labeling (B and D) at 8 dpi from IEPy and EEPy parasagittal sections. There was a higher intensity of anti-CD3 immunolabeling and tomato lectin histochemical staining in EEPy as compared with IEPy animals. In EEPy, the higher level of CD3 immunostaining was observed in the septum whereas tomato lectin histochemical staining seemed to be more intense in the hippocampal fimbria, confirming that *Lycopersicum esculentum* not only labeled microglia and T lymphocytes but also other inflammatory cells with polylactosamine structures such as monocytes [21], [22]. A striking inverse correlation between the intensity of Piry virus immunolabeling (Figure 3B and D, inserts) and anti-CD3 (Figure 3A and C) or tomato lectin (Figure 3B and D) staining was also observed. Note that the morphology of labeled cells in Fig. 3Bi and Di appear very different. Since they are both samples labeled with tomato-lectin it is possible that they are indeed different cells. New experiments will be necessary to clarify the nature of these cellular infiltrates under different environments. Although we have performed CD3, CD8 and CD43 immunolabeling in all experimental groups we have not found any labeling in the uninfected subjects.

**Figure 4:**
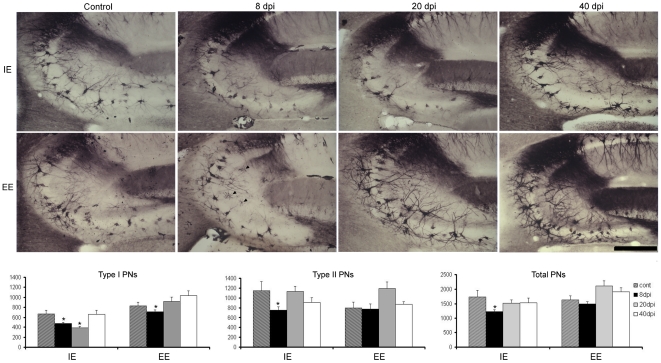
Perineuronal net (PN) damage revealed after lectin-histochemical staining with *Wisteria floribunda* agglutinin. Top and middle: photomicrographs of PNs of CA2–CA3 hippocampal fields from representative parasagittal sections of control and Piry virus–infected mice at 8, 20, and 40 dpi maintained in impoverished (IE) or enriched (EE) environments. Bottom: total numbers of PNs and respective standard errors bars (s.e.m.) for CA3 estimations in control and infected animals at 8, 20, and 40 dpi. Type I PNs (left); type II PNs (center); total PNs (right). Two-way ANOVA for type I revealed that perineuronal nets estimations were influenced by the environment (F = 15.41; *p* = 0.0004) and survival time (F = 3.12; *p* = 0.039) with a non-significant interaction between these variables (F = 0.11; *p* = 0.95). Scale bar: 25 µm.

A specialized feature of the extracellular matrix, known as the perineuronal net, contains glycosaminoglycans (GAGs) and these are targeted by a variety of viruses on the surface of host cells [9]. PNs can be promptly revealed after lectin-histochemical staining with *Wisteria floribunda* agglutinin [23] Figure 4 presents the results of specialized extracellular matrix damage Photomicrographs were taken from the animal that best represented the average values of stereological counts. The limits of CA2 and CA3 hippocampal fields were clearly distinguished by a histochemical pattern in which a darker neuropil indicated the CA2 field frontiers. Figure 4 compares the effect of viral infection on the reduction of PNs at different time windows in both IEPy (first row) and EEPy (second row) animals and presents the results of stereological estimations (third row). The larger type I PNs (perisomatic and peridendritic) were clearly affected by the encephalitis both in IEPy and EEPy animals at 8 dpi. Total PN estimations showed that they were reduced only at 8 dpi in IEPy mice (one-way ANOVA, Bonferroni test *p*<0.05), but not in EEPy animals as compared to uninfected respective controls. However, type I and II PNs differed in their response; in IEPy mice, type I remained reduced until 20 dpi but recovered to control levels at 40 dpi, whereas in EEPy animals, type I PNs were significantly reduced only at 8 dpi (one-way ANOVA, Bonferroni test *p*<0.05). Type II PNs and totals (Type I plus Type II) were reduced in number only at 8 dpi in IEPy (one-way ANOVA, Bonferroni test *p*<0.05) but not in EEPy animals.

Tables S1, S2 and S3 presented as supplementary material correspond to stereological estimations of microglia and PNs at 8, 20 and 40 dpi respectively.

Two-way ANOVA revealed a significant influence of environmental conditions (F = 20.71; *p*<0.0001) and survival time (F = 38.73; *p*<0.0001) after infection on the total number of microglia in CA3, with a significant interaction between these variables (F = 4.18; *p* = 0.0138) whereas the number of type I PNs was influenced by the environment (F = 15.41; *p* = 0.0004) and survival time after infection (F = 3.12; *p* = 0.039) with a nonsignificant interaction between these variables (F = 0.11; *p* = 0.95). However two-way ANOVA applied to the type II PNs revealed no significant differences between the experimental groups, suggesting that type I PNs are more sensitive to the viral infection and that the extracellular matrix regeneration is faster in EE than in IE animals.

Finally, the number of CA3 neurons did not differ between IEPy and EEPy mice or compared to their respective control groups at 20 dpi, regardless of environmental conditions (Table S4; IEcont: 35742±1920; IEPy: 37244±8480; EEcont: 35104±3438; EEPy: 33496±4825; mean ± s.d.; one-way ANOVA, *p*>0.05), indicating that microgliosis, PNs reduction, and behavioral changes were not associated with neuronal death.

The variance introduced by methodological procedures was, in most cases, less than 50% of the observed group variance, giving a ratio of CE^2^/CV^2^<0.5, where CE corresponds to the coeficient of error introduced by the stereological procedures and CV is the coeficient of variation [24]. In the cases of experimental groups that did not follow this rule, a CE^2^/CV^2^ ratio >0.5 was not indicative of a large variance introduced by stereological analysis. In this exception, both the CV and CE were low, and the general rule was neither meaningful nor practical to follow [24].

It has been previously demonstrated a direct correlation between microglial activation and extracelular matrix damage [25]. Table S5 list the coefficients of correlations between microglial numbers and different types of PNs estimations by the optical fractionator at different time windows. A significant inverse correlation was detected between the number of microglias and the number of type I PNs at 8 dpi (p = 0.001; coeficient of correlation  = 0.72) and 20 dpi (p = 0.008; coeficient of correlation = 0.63) suggesting that the inflammatory response may contribute to the observed PNs damage during Piry viral encephalitis.

### Behavioral changes

**Figure 5:**
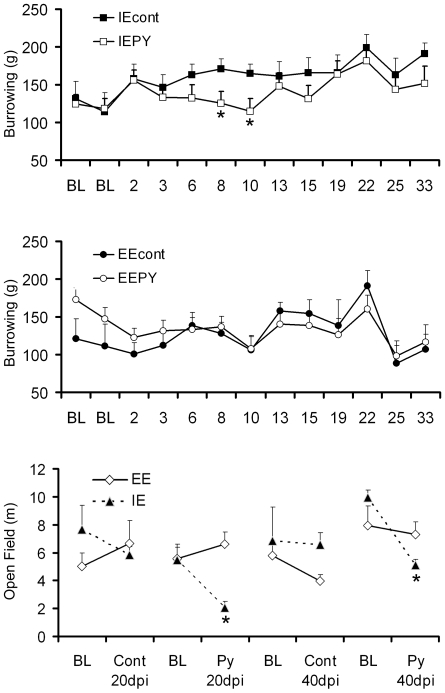
Sickness behavior. Graphic representation of the behavioral changes with respective standard error bars (s.e.m) in the albino Swiss mouse model of Piry virus encephalitis. A, amount of borrowed food in the burrowing test in IE animals; B, amount of borrowed food in the burrowing test in EE animals; C, travel distance in the open field test. On average, IEPy animals started to burrow less food at 8 dpi and recovered to control levels at 13 dpi. Among IEPy animals, open-field tests appeared altered at 20 dpi and remained so at 40 dpi. EEPy presented no significant changes in burrowing and open-field tests. BL: baseline * *p*<0.05, one-way ANOVA, Bonferroni *a priori* test. IEcont, EEcont: impoverished and enriched environment control groups, IEPy, EEPy: impoverished and enriched environment infected groups.

The first behavioral changes became apparent at 8 dpi, when IEPy animals burrowed significantly less food than IE controls and continued until 13 dpi, when burrowing activity recovered to control levels (Figure 5, top). No significant differences were detected in burrowing between EEPy and EE control animals (Figure 5, middle). Locomotor and exploratory activities assessed by open-field tests appeared altered later in the disease (20 dpi) and remained so at 40 dpi in IEPy animals, whereas no significant change was observed in EEPy mice (Figure 5, bottom). No significant differences were detected in the dark/light box or elevated plus maze tests.

## Discussion

In the present report, as a model to study encephalitis outcomes in adult albino Swiss mice, we selected the Piry virus, a member of a group of RNA South American viruses [26], [27], found in Brazil, that causes febrile disease in humans [28], [29] and encephalitis in a neonate murine model [19], [30], [31]. In mice housed under IE or EE conditions, we induced viral encephalitis by intranasal inoculation of Piry virus–infected brain homogenate and correlated neuropathological features quantified using a stereologically based unbiased method with behavioral changes, comparing the outcomes with those of animals inoculated with uninfected brain homogenate.

Although a few reports describe application of stereological methods to quantify neuropathological features in correlation with behavioral changes to study encephalitis [32], [33], [34], none have investigated the effect of an EE on viral encephalitis. Indeed, only two studies have investigated the effect of an EE on brain infections using experimental models [35], [36], but their approach to quantifying neuropathological changes was not based on unbiased stereological estimations. As a result, quantitative associations between neuropathological features and behavioral changes in murine models of encephalitis and the effect of EE have previously not been firmly established.

To quantify neuropathological changes, we applied the optical fractionator, an accurate method of quantification combining properties of an optical dissector and the fractionator that has been used in a variety of studies to determine cell numbers in multiple brain regions [37], [38]. The optical fractionator is unaffected by histological changes, shrinkage, or damage-induced expansion by injury, an issue of particular importance when studying brain diseases [37], [39], [40].

With these tools, we have shown here that the behavioral and neuropathological consequences of Piry virus encephalitis are more severe in animals living under IE conditions in comparison with encephalitis outcomes in mice housed under EE conditions.

### Microglial activation and neuronal death

The occurrence of neuronal death after encephalitis induced by rhabdoviruses remains controversial [41], [42], [43], [44], but in general, when it is unequivocally detected, it seems to be a microglial-mediated event (e.g. [45],[46]). To investigate possible losses of neuronal numbers in correlation with microglial activation after Piry virus encephalitis, we used NeuN as a selective immunohistochemical marker. The microglial host response was more intense and generalized in the brain parenchyma at 8 dpi compared to 20 or 40 dpi. Viral neuroinvasion mainly included the olfactory pathways, septum, amygdala, and ventral CA3 and polymorphic layer of the ventral dentate gyrus. There was an association between the intensity of viral antigen labeling in the parenchyma, a higher number of microglias, and a greater reduction of PNs, especially type I, without significant neuronal death. In addition, the intense immunolabeling of T lymphocytes in the brain was associated with environmental enrichment suggesting a higher mobilization of these cells to the brain parenchyma during Piry viral encephalitis. These findings suggest that microglia activation and extracellular matrix damage may be key factors in the pathogenesis of Piry encephalitis and that an EE differentially regulates microglial activation, increases T cell infiltration, preserves the extracellular matrix, and promotes faster virus clearance from the brain.

### Neuroinvasion, the anti-inflammatory environment, and virus clearance

In the albino Swiss mouse model of viral encephalitis, microglial activation revealed by tomato lectin histochemistry occurred relatively early during disease progression (8 dpi) when the first behavioral changes became apparent. Tomato lectin also binds to monocytes and B and T infiltrating lymphocytes [21], [22], revealing a conspicuous accumulation of lymphocytes in virus-infected areas. In the present report, these small rounded tomato lectin-positive cells, morphologically distinct from ameboid microglia, accumulated in infected areas in greater concentrations in animals housed under EE conditions when compared to those housed in impoverished conditions. Of importance, no apparent difference was found between uninfected animals in EE and IE conditions in terms of the distribution of T cells in the brain parenchyma. A significant number of CD3- and some CD8- and CD43-positive cells were found in the same regions (e.g., fimbria, septum) where tomato lectin-positive cells were detected. These results are compatible with previous data on VSV encephalitis [15], [47].

Intranasal application of VSV induces acute encephalitis characterized by a pronounced myeloid and T cell infiltration with two distinct phagocytic populations regulating VSV encephalitis but not virus clearance [48]. VSV encephalitis is characterized by a pronounced infiltrate of myeloid cells (CD45^+^, CD11b^+^) and CD8^+^ T cells containing a subset specific to the immunodominant VSV nuclear protein epitope [15]. However, because ablation of peripheral macrophages does not impair VSV encephalitis or viral clearance from the brain but depletion of splenic marginal dendritic cells impairs this response and enhances morbidity/mortality [48], it is tempting to speculate that these dendritic cells may also be increased in EEPy as compared to IEPy animals. Another possibility is that the EE may induce NK cell activity previously described as absent in a VSV encephalitis murine model maintained in standard cages [15]. In line with these views, voluntary exercise such as that observed in an EE increases the number of blood dendritic cells [49], [50], [51], [52] and NK cells [6]. Because we did not use selective markers for other immune cells such as recruited monocytes/macrophages [53], dendritic cells [15], or NK cells [54], it remains to be confirmed whether these cells are associated or not with the immune response induced by Piry virus infection and whether or not EE affects their distribution and number.

Enriched housing conditions mitigate the inflammatory response after stroke [55], reduce the imbalances between contra- and ipsilateral reactive astrocytosis in a rat model of chronic inflammatory pain [56], induce beneficial astrogliogenesis to preserve the nigrostriatal system against 6-OHDA-induced toxicity [57], and alter the inflammatory response in the hippocampus of a transgenic mouse model of Alzheimer disease, TG2576 [58]. More recent work indicates that exercise training improves macrophage innate immune function in a beta(2)AR-dependent and -independent manner with lipopolysaccharide-stimulated nitric oxide and proinflammatory cytokine production in macrophages from trained mice being markedly higher than those from control mice [59].

In the present report, we expanded these observations, demonstrating that environmental conditions significantly affect disease outcomes in sublethal encephalitis. Because previous work had revealed that Piry virus neuroinvasion targets hippocampal fields [19] and that *Wisteria floribunda* histochemical staining conspicuously defines the architectonic limits of the hippocampal fields in adult mice [23], we chose to limit the quantitative neuropathological analysis to CA3. EE reduced the neuroinflammatory response and extracellular matrix damage in CA3. These findings are in agreement with those of previous reports suggesting that regular exercise provokes anti-inflammatory cytokines [60]. These previous data found an association between IL-6 and acute exercise, which was followed by stimulation of the production of anti-inflammatory cytokines and cytokine inhibitors, such as IL-1ra and IL-10, as well as an increase in IL-6 receptors during regular exercise. This favorable anti-inflammatory environment may explain the beneficial effects of exercise on acute neuroinflammation associated with the EE, as described in the present report.

As the disease resolved (20 and 40 dpi), the increased number of microglial cells in all infected animals and migratory CD3-positive cells in EE mice decreased and approached control levels after virus clearance. In line with these findings, the number of PNs increased in the infected animals in an inverse proportion to the activated microglial numbers in CA3, recovering the integrity of the extracellular matrix and normal behavior. These events were independent of the number of neurons in CA3 that remained unaltered during the disease course.

### Behavioral outcomes

Morphologically activated microglia following Piry virus neuroinvasion were observed mainly in the olfactory pathways, septal region, amygdala, ventral CA3, and the polymorphic layer of the ventral dentate gyrus. Because septal damage mimics the effects of both dorsal and ventral hippocampal lesions [61], we selected ventral and dorsal hippocampal-dependent tasks to investigate behavioral changes [62], [63], [64], [65]. Performances on all of these tasks are altered after hippocampal damage [65], [66], [67].

Burrowing changes started at 8 dpi and recovered to control levels at 13 dpi. Open-field tests presented significant differences between IEPy and IE control mice at 20 dpi and remained altered after 40 dpi. In contrast, animals housed under EE conditions had no significant differences in these tests. Assuming that the open field detected possible anxiety-like behavior associated with ventral hippocampus damage [64], [65], [68], that burrowing activity detects selective damage of the dorsal hippocampus [62], [63], [69], and that there was no apparent virus immunolabeling in the dorsal hippocampus, burrowing changes may be associated with septal damage [61].

In the murine model of VSV encephalitis, reactive astrocytosis and microglial activation occur relatively early in the disease [15], [70]. As the disease progresses, these non-neuronal cells proliferate with an increasing effect on the extracellular matrix [71]. In the present report, microgliosis and a reduction in type I PNs in CA3 of IE mice were significantly correlated at 8 and 20 dpi, suggesting that the inflammatory response may be related to extracellular matrix damage. As soon as microglia activation was reduced during the disease recovery process, type I PNs started to recover up to control levels. Because the integrity of the extracellular matrix is important for long-term potentiation in the hippocampus [72], it may be possible that the observed type I perineuronal losses correlated, at least in part, with the transient behavioral changes observed with Piry virus encephalitis.

### Conclusion

We report for the first time that an EE induces less intense behavioral changes, a lesser degree of microgliosis, a smaller reduction in the number of PNs, a higher degree of T cell infiltration, and faster virus clearance and disease resolution when compared to animals exposed to impoverished housing. We also demonstrated that nasal instillation of Piry-infected brain homogenate into adult albino Swiss mice induces (i) encephalitis with neuroinvasion, mainly of the olfactory pathways, septum, amygdala, and ventral hippocampus; and that (ii) the infection leads to an increase in CA3 microglial number and reduction of the PNs at 8 dpi when behavioral changes first appear, without changes in the number of neurons.

The mechanisms of neuronal protection that are activated during the faster clearance of the viruses from the brains of EE animals remain to be investigated. Detailed cellular and molecular analysis built on these observations, including characterization of the inflammatory cells mobilized to the brain parenchyma as well as viral neuroninvasion and clearance mechanisms, may delineate the pathopysiological basis of these events, improving our understanding of non-pharmacological treatment of neurological disorders.

## Materials and Methods

All procedures were submitted to and approved by the institutional animal care committee of the Federal University of Pará. We used 59 2-week-old albino Swiss mice obtained from the Animal Care Facility of Instituto Evandro Chagas and handled in accordance with the “Principles of Laboratory Animal Care” (NIH).

### Experimental groups and inoculation

Suckling mice were intracerebrally infected with 10 µl of infected brain suspension, anesthetized, and perfused after becoming sick. The brains were histologically processed for immunohistochemistry procedures using specific anti-virus antibodies. Virus-containing brain homogenates were obtained as follows. First, 0.02 ml of each viral suspension was intracerebrally inoculated into newborn mice, and the animals were observed daily. Upon presenting with clinical signs, the animals were sacrificed and immediately stored at −70°C. Later, the brain tissue (0.2 g/animal) was macerated and mixed with 1.8 ml phosphate-buffered saline (PBS) containing 100 U/ml penicillin and 100 mg/ml streptomycin. The suspension was cleared by centrifugation at 10,000×g for 15 min at 4°C. Virus titration was carried out by intracerebral inoculation of newborn mice with 0.02 ml of serial 10-fold dilutions of the viral suspensions in PBS, and LD50 values were calculated by the method of Reed and Muench. The Piry virus titers in the sample (LD50/0.02 ml) were 8.0 Log10. The choice of viral concentration obeyed the criterion of using a non-lethal dose that still induces a sublethal encephalitis in adult mice, achieved with a dilution of 1∶100,000 of the sample titrated.

To confirm the presence of the virus in the brains of animals used to prepare infected brain homogenate, some of the animals were processed to obtain ultrathin sections for analysis with a Zeiss EM 900 transmission electron microscope, as described elsewhere [73]. In brief, samples obtained from neonate brains after perfusion and craniotomy were cut into small fragments and immersed for 2 h in a fixative solution containing 2.5% glutaraldehyde in 0.1 M phosphate buffer, pH 7.2, at room temperature. After primary fixation, brains were immersed in 0.1 M cacodylate buffer and post-fixed in a solution containing 1% osmium tetroxide, 0.8% potassium ferrocyanide, and 5 mM CaCl_2_ at room temperature in the same buffer. Sections were en bloc stained with 2% uranyl acetate in 25% acetone, dehydrated in graded acetone concentrations, and embedded in EMbed-812 (Electron Microscopy Sciences, Fort Washington, PA, USA). Ultrathin sections were obtained with a Reichert/Leica Ultracut S ultramicrotome (Leica Microsystems, Bannockburn, IL, USA) and stained with aqueous uranyl acetate and lead citrate before examination. Figure S2 shows a representative electronmicrograph of a neonate cortical region obtained by transmission electron microscopy to illustrate the typical bullet morphology of the Piry virus in the neonate brain used to prepare the infected brain homogenate.

### Behavioral analysis

The mice were kept in an impoverished environment (IE, n = 31) or enriched environment (EE, n = 28) for three months and then submitted to the following tests: open field, burrowing, dark/light box, and elevated plus maze. Enriched environmental conditions corresponded to plastic cages (32 cm×39 cm×16.5 cm) with chopped rice straw on the floor and equipped with rod bridges, tunnels, running wheels, and toys made of plastic, wood, or metal with different forms and colors that were changed every week. The IE cages corresponded to plastic cages with the same dimensions and chopped rice straw on the floor but without equipment or toys. Each cage housed 12–15 mice. All mice had free access to water and food, and 12-h dark and light cycles were maintained. Tests occurred during the light cycle.

**Burrowing:** Two hours daily (from 09:00 to 11:00 h) for 3 consecutive days before inoculation and from post-inoculation days 2 to 35, all animals were placed in individual plastic cages (32 cm×39 cm×16.5 cm) containing a PVC tube (20 cm long, 7.2 cm diameter) filled with 250 g of normal diet food pellets. The open end was supported 3 cm above the floor. After the testing period, the remaining food in the cylinders was weighed and the mice returned to their collective cage [66].

**Open field:** The apparatus consisted of a gray PVC box (30 cm×30 cm×40 cm) with the floor divided into 10-cm squares. For 3 consecutive days before inoculation, each animal was placed in one corner and kept in the apparatus for 5 min. One meter above the open field, a video camera connected to a computer recorded each training session for later analysis by Any-Maze software (Stöelting). The following parameters were analyzed: distance traveled (m), mean speed (m/s), and immobility time (s). After each section, the open field was cleaned with 70% ethanol.

**Elevated plus maze:** The elevated plus maze consisted of two open (30 cm×5 cm, no border) and two closed arms (30 cm×5 cm, surrounded by a 15-cm wall) placed in opposite positions and connected by a central platform (5 cm×5 cm). The apparatus was elevated 45 cm from the floor. Each animal was placed in the central platform facing one of the open arms and left there for 5 min. The test was performed over 2 consecutive days, and each animal had one session per day. All sessions were recorded and the following parameters analyzed by Any-Maze software (Stöelting): number of entries, time remained, and distance traveled in the open and closed arms. The program was set to define an arm entry when the center of the body of the animal entered the new area. For the sake of comparative analysis, the parameters were expressed as contrast values between the open and closed arms according to the following equation: C = (c−o)/(c+o), where C represents the contrast index and c and o correspond to parameters for the closed and open arms, respectively. The application of the contrast formula normalized the scale and allowed us to compare the anxiety-like behavior between groups with distinct patterns of locomotor activity, measuring possible differences more accurately.

**Dark/light box:** We adapted the dark/light box test from a previously published protocol [74]. The apparatus consisted of an open-topped rectangular box (45 cm×27 cm×30 cm high) divided into small (18 cm×27 cm) and large (27 cm×27 cm) areas with an open door (7.5 cm×7.5 cm) located in the center of the partition at floor level. The small compartment was painted black and kept at a dim light level (0.38 cd/m^2^), whereas the large compartment was painted white and brightly illuminated (36.4 cd/m^2^). The test was performed in a quiet, dark room. All sessions were recorded with a webcam and the following parameters were analyzed by Any-Maze software (Stoelting): number of entries, time spent, and distance traveled in the light compartment. The program was set to define a compartment entry when the center of the body of the animal entered the new area. The mice were kept in this room at least 1 h before the test. To reduce any neophobic response to the test situation, the light/dark compartments were previously soiled by mice other than those used during the test. Mice were always tested in a soiled apparatus, and there was no cleaning between trials. Naive mice were placed individually in the middle of the light area facing away from the opening. The images of the light compartment were recorded during a 5-min test.

For 6 consecutive days before inoculation, all mice were submitted to open field (days 1 to 3), LCE (days 4 and 5), and dark/light box (day 6) to obtain a baseline curve for these tests. After 20 days post-inoculation (dpi), 29 animals were submitted to the same tests, and the remaining mice (n = 30) were tested with the same protocol after 40 dpi.

After behavioral tests, all animals were anesthetized with intraperitoneal 2,2,2-tribromoethanol 1% (0.01 ml/g of body weight) and intra-nasally challenged with 5 µl viral suspension (10^−5^ v/v in 100 U/ml penicillin, 100 µg/ml streptomycin) or normal brain homogenate as a control (10% v/v in 100 U/ml penicillin, 100 µg/ml streptomycin).

After recovering, animals were housed in enriched or standard plastic cages (32 cm×39 cm×16.5 cm) and maintained in the Instituto Evandro Chagas (Belém – PA) animal house, where they remained until the end of the experiments. All animals were kept in the care facility room at 23±2°C with *ad libitum* access to food and water and with a 12-h light/dark cycle.

All mice were tested again (open field, burrowing, dark/light box, and elevated plus maze) after 20 dpi (IEPy, experimental, n = 10; IE control, n = 7; EEPy, n = 5; EE control, n = 7) or 40 dpi (IEPy, n = 8; IE control, n = 6; EEPy, n = 11; EE control, n = 5).

### Neuropathology

When each animal reached the survival time of its group after behavioral tests, the mice were weighed and anesthetized with intraperitoneal 2,2,2-tribromoethanol (0.04 ml/g of body weight) and transcardially perfused with heparinized saline, followed by 4% paraformaldehyde in 0.1 M phosphate buffer (pH 7.2–7.4). Alternate series of sections (70 µm thickness) obtained using a Vibratome (Micron) were immunolabeled with polyclonal antibody for Piry virus antigens, monoclonal antibody for glial fibrillary acid protein (GFAP) to detect astrocytes, or monoclonal antibody for CD3- and CD8-positive T cells or histochemically reacted to detect the biotinylated lectins *Lycopersicum esculentum* (activated microglia) and *Wisteria floribunda* (PNs) supplied by Vector Laboratories (CA, USA). All chemicals used in this investigation were supplied by Sigma-Aldrich (Poole, UK) or Vector Laboratories (CA, USA), and the GFAP and Piry primary antibodies were from Chemicon (CA, USA) and Instituto Evandro Chagas (PA, Brazil), respectively.

### Immunohistochemistry and histochemistry

To assess the distribution of Piry viral antigens, CD8 T cells, and astrocytes in the mouse brain at the different time points, immunohistochemistry was performed on all infected and in five control animals. Specific antibodies against Piry virus species were produced by the Unit of Arbovirus and Hemorrhagic Fevers at the Instituto Evandro Chagas, as described elsewhere [73], [75]. In brief, free-floating sections were rinsed in 0.1 M phosphate buffer and placed in a solution of 0.2 M boric acid (pH 9.0) at 70°C for 1 h for antigen retrieval. After being rinsed in 0.1 M PBS with 5% Triton X-100, sections were incubated in a solution of methanol and 0.3% hydrogen peroxide for 10 min. After washing in PBS, the Mouse-on-Mouse (MOM) Blocking Kit (M.O.M. kit, Vector Laboratories, Burlingame, CA, USA) was used as follows: MOM IgG blocking for 1 h, primary antibody for 72 h (GFAP 1∶800, Chemicon, CA, USA; anti-CD3 T lymphocytes 1∶1000, MCA500G and anti-CD 43 MCA1096 1∶200 AbD Serotec, Oxford, England, UK; anti-CD8 T lymphocytes 1∶100, Cod 140083 eBioscience, San Diego, CA, USA; anti-Piry 1∶20, Instituto Evandro Chagas, PA, Brazil), and MOM Biotinylated Anti-Mouse IgG Reagent for 12 h. Sections were washed in PBS and transferred to avidin-biotin-peroxidase complex (ABC) (Vector Laboratories) solution for 1 h, washed again before incubation in 0.2 M acetate buffer (pH 6.0) for 5 min, and revealed in GND solution (diaminobenzidine 0.6 mg/ml, ammonium nickel chloride 2.5 mg/ml, and glucose oxidase). All steps were carried out under gentle and constant agitation. All chemicals used in this investigation were supplied by Sigma-Aldrich (Poole, UK). As a negative control, normal horse serum was added to some slides instead of primary antibody for each antibody used as a cell marker and processed for immunohistochemistry as previously described.

Other sections were used to detect microglia activation and PNs by histochemistry with biotinylated *Lycopersicum esculentum* (tomato) lectin and biotinylated *Wisteria floribunda* lectin, respectively, according to manufacturers' instructions with small adaptations. Briefly, sections were rinsed in PBS with 5% Triton X 100 for 20 min and transferred to a solution of methanol and 0.1% hydrogen peroxidase for 10 min. After being washed in PBS, the sections were incubated in the lectin solutions (*Lycopersicum esculentum*, 6 µg/ml; *Wisteria floribunda*, 9 µg/ml) overnight at 4°C, placed in ABC solution for 1 h, and revealed with GND solution following the same protocol previously described for immunohistochemistry. After this process, all sections reacted for *Lycopersicum esculentum* were counterstained in 0.5% cresyl violet.

### Microscopy and optical fractionator

Details of the optical fractionator methodology are described under Stereological Procedures in the online supporting material (see Text S1). Tables S6–S8 present the stereological parameters and counting protocol and results for microglia and PNs in CA3.

**Area and objects of interest:** The limits of CA2/CA3 were defined by architectonic differences in the neuropil after *Wisteria floribunda* histochemistry, in which CA2 appears darker than CA3 (Figure S3). The PN counting procedure included two types of nets: type I corresponding to perisomatic and peridendritic nets, including secondary tertiary branches, and type II corresponding to perisomatic and faint primary dendrites (Figure S3).

We used histochemical reactions to reveal biotinylated *Lycopersicum esculentum* and *Wisteria floribunda* as markers of activated microglia and PNs. Detection of poly-N-acetyl lactosamine residues with biotinylated *Lycopersicum esculentum* (tomato lectin) also reveals T cell distribution and quiescent microglia [20], [21]. *Wisteria floribunda* histochemistry selectively labels the n-acetyl-galactosamine β1 residues of glycoproteins, revealing PNs within the extracellular matrix and conspicuously defining the architectonic limits and layers of mice hippocampal fields CA3/CA2/CA1 [23]. Neurons were selectively labeled by immunohistochemistry to detect NeuN, a nuclear protein present in the vast majority of post-mitotic neuronal cells in vertebrates [76]. In the mouse hippocampus, where the area of interest for the present report is located, an extensive series of studies has already established NeuN as selective neuronal marker [77], [78].

**Photomicrographic documentation and processing:** To obtain digital photomicrographs, we used a digital camera (Microfire, Optronics, CA, USA) coupled to a Nikon microscope (Optiphot-2, NY, USA). Digital photomicrographs were processed using Adobe Photoshop 7.0.1 C.S.2 software (San Jose, CA, USA) for scaling and adjusting the levels of brightness and contrast. For the figures, selected pictures were taken of sections from the animals in each experimental group with the total number of objects of interest nearest the mean value of each region of interest.

### Statistical analyses

All groups were compared using parametric statistical analysis, one-way ANOVA, Bonferroni *a priori* test, or two-way ANOVA followed by Bonferroni post-tests, with differences between groups accepted as significant at a 95% confidence level (*p*<0.05).

## Supporting Information

Text S1 **Stereological Procedures**
(RTF)

Figure S1 **Cellular infection of Piry virus in EEPy and IEPy.** Differential degree of Piry virus cellular infection in IE and EE infected subjects. Note less immunolabeled cells in EEPy as compared to IEPy subject. OB: olfactory bulb; DG: dentate gyrus; CA3: Ammonis Cornus 3. Scale bar: 250 µm.
(TIF)

Figure S2 **Electron micrograph of Piry virus in the cerebral cortex.** Electron micrograph of Piry virus in the cerebral cortex of neonate albino Swiss mice used to prepare infected brain homogenate. Note the typical bullet morphology of this Rhabdovirus species (arrow heads).
(TIF)

Figure S3 **CA3 limits and perineuronal net types.** Photomicrographs of histochemically reacted parasagittal sections of the architectonic limits of CA3 (low power) and types of perineuronal nets (high power). The CA3 pyramidal cell layer was outlined after histochemical reactions for *Wisteria floribunda* lectin (A) and immunohistochemistry for NeuN (not illustrated). Note that the *Wisteria floribunda* histochemical reaction labeled two types of perineuronal nets, indicated in the picture as type I and II. Arrows point to type II and asterisk to type I perineuronal nets. Scale bars: low power 250 µm; high power 25 µm.
(TIF)

Table S1 **Microglial and perineuronal net estimations at 8 d post inoculation.**
(DOC)

Table S2 **Microglial and perineuronal net estimations at 20 d post inoculation.**
(DOC)

Table S3 **Microglial and perineuronal net estimations at 40 d post inoculation.**
(DOC)

Table S4 **Neuronal estimations at 20 d post inoculation.**
(DOC)

Table S5 **Correlations between microglial activation and extracellular matrix damage.**
(DOC)

Table S6 **Stereological parameters for microglial estimations and counted markers.**
(DOC)

Table S7 **Stereological parameters for perineuronal net estimations and counted markers.**
(DOC)

Table S8 **Stereological parameters for neuronal estimations and counted markers.**
(DOC)
