# Supplementary material for: Influence of Enriched Environment on Viral Encephalitis Outcomes: Behavioral and Neuropathological Changes in Albino Swiss Mice
Source: PLoS One. 2011 Jan 11;6(1):e15597. doi: 10.1371/journal.pone.0015597 (PMC3019164; doi:10.1371/journal.pone.0015597)
Supplement: Text S1 — Stereological Procedures (RTF) [file pone.0015597.s001.rtf]

Supporting Text S1
Stereological procedures
At all levels in the histological sections, we delineated the layers of the CA3 region, digitizing directly from sections with a low resolution, 3.2× objective on a Optiphot-2 microscope (Nikon, Japan) equipped with a motorized stage (MAC200, Ludl Electronic Products, Hawthorne, NY, USA). This system was coupled to a computer that ran Stereoinvestigator software (MicroBrightField, Williston, VT, USA), used to store and analyze x, y, and z coordinates of digitized points. In order to detect and count the objects of interest unambiguously with the dissector probe, the low resolution objective was replaced with a high resolution, 60× oil immersion planapochromatic objective (NIKON, NA 1.4). Thus, all stereological estimations started with the delineation of the region of interest in horizontal sections, where the layers and limits of the CA3 were unambiguously identified and outlined. The border between the polymorphic layer and the CA3 region was arbitrarily defined in horizontal sections with a straight line that connected the tip of the pyramidal cell layer of the CA3 with the two tips of the granular cell layer. At each counting site, the thickness of the section was carefully assessed with the high resolution objective, and the fine focus of the microscope was used to define the immediate layers at the top, middle, and bottom of the section. Because both the thickness and the distribution of cells in the section were variable, the total number of objects of interest was weighted with the section thickness. All objects that came into focus inside the counting frame were counted and added to the total number of markers, provided they were entirely within the counting frame or intersected the acceptance line without touching the rejection line (Gundersen & Jensen, 1987). The counting boxes were randomly, systematically placed within a grid. Tables S6-S8 present the experimental parameters and average counting results obtained with the optical fractionator. With this optical fractionation of sections, we were able to determine the CA3 number of markers (∑Q-) in adult female albino Swiss mice. The grid sizes were adapted to achieve an acceptable coefficient of error (CE). For the CE of the total cell counts for each subject in the present study, we adopted the one-stage systematic sampling procedure (Scheaffer CE) used previously and validated elsewhere (Glaser & Wilson, 1998). The level of acceptable errors for stereological estimations was defined as the ratio between the intrinsic error introduced by the methodology and the coefficient of the variation (Glaser & Wilson, 1998; Slomianka & West, 2005). The CE expresses the accuracy of the cell number estimates, and a value of CE ≤ 0.05 was deemed appropriate for the present study, because variance introduced by the estimation procedure contributed little to the observed group variance (Glaser & Wilson, 1998; Slomianka & West, 2005). The experimental parameters were established in pilot experiments and uniformly applied to all animals. 
Tables S1-S3 present optical fractionator average counting results of objects of interest (microglia and perineuronal nets) in CA3 of each experimental group at 8, 20 and 40 days respectively. Table S4 present individual unilateral neuron numbers (n) with coefficients of error (CE) for CA3 in adult female albino Swiss mice 20 d after nasal instillation of Piry (PY)-infected or normal brain homogenates.
The stereological based unbiased analysis of the objects of interest in CA3 with the optical fractionator method detected significant differences in the inflammatory response (number of microglias) and specialized extracellular matrix changes following induced encephalitis after environmental enrichment.
Table S5 represents coefficient of correlations between microglial numbers and different types of perineuronal net estimations by optical fractionator at different time windows.  Note that good correlation was obtained only with type I perineuronal nets.
