# Supplementary figures and images for: Influence of Enriched Environment on Viral Encephalitis Outcomes: Behavioral and Neuropathological Changes in Albino Swiss Mice
Source: PLoS One. 2011 Jan 11;6(1):e15597. doi: 10.1371/journal.pone.0015597 (PMC3019164; doi:10.1371/journal.pone.0015597)

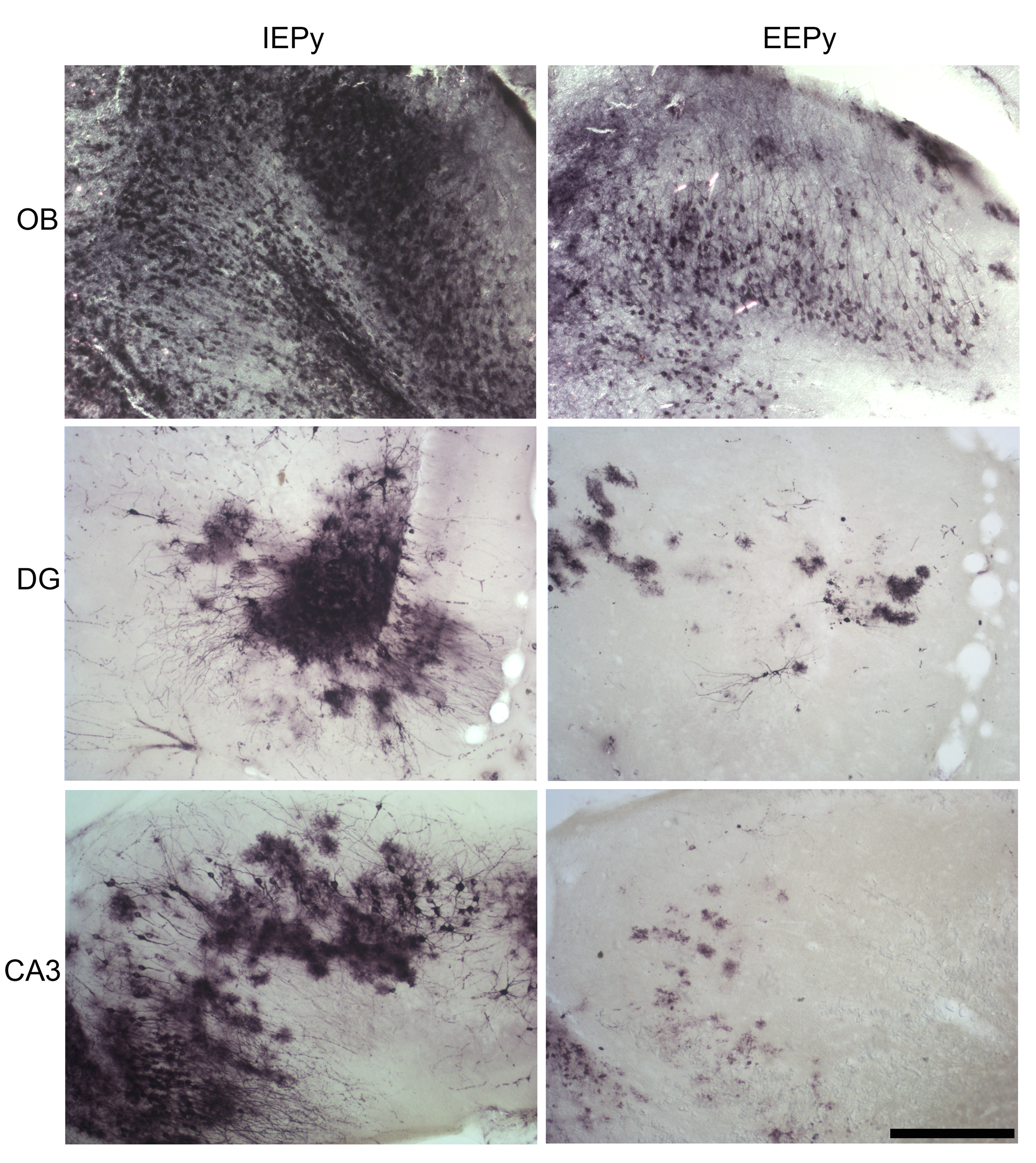

Supplement: Figure S1 — Cellular infection of Piry virus in EEPy and IEPy. Differential degree of Piry virus cellular infection in IE and EE infected subjects. Note less immunolabeled cells in EEPy as compared to IEPy subject. OB: olfactory bulb; DG: dentate gyrus; CA3: Ammonis Cornus 3. Scale bar: 250 µm. (TIF) [file pone.0015597.s002.tif]

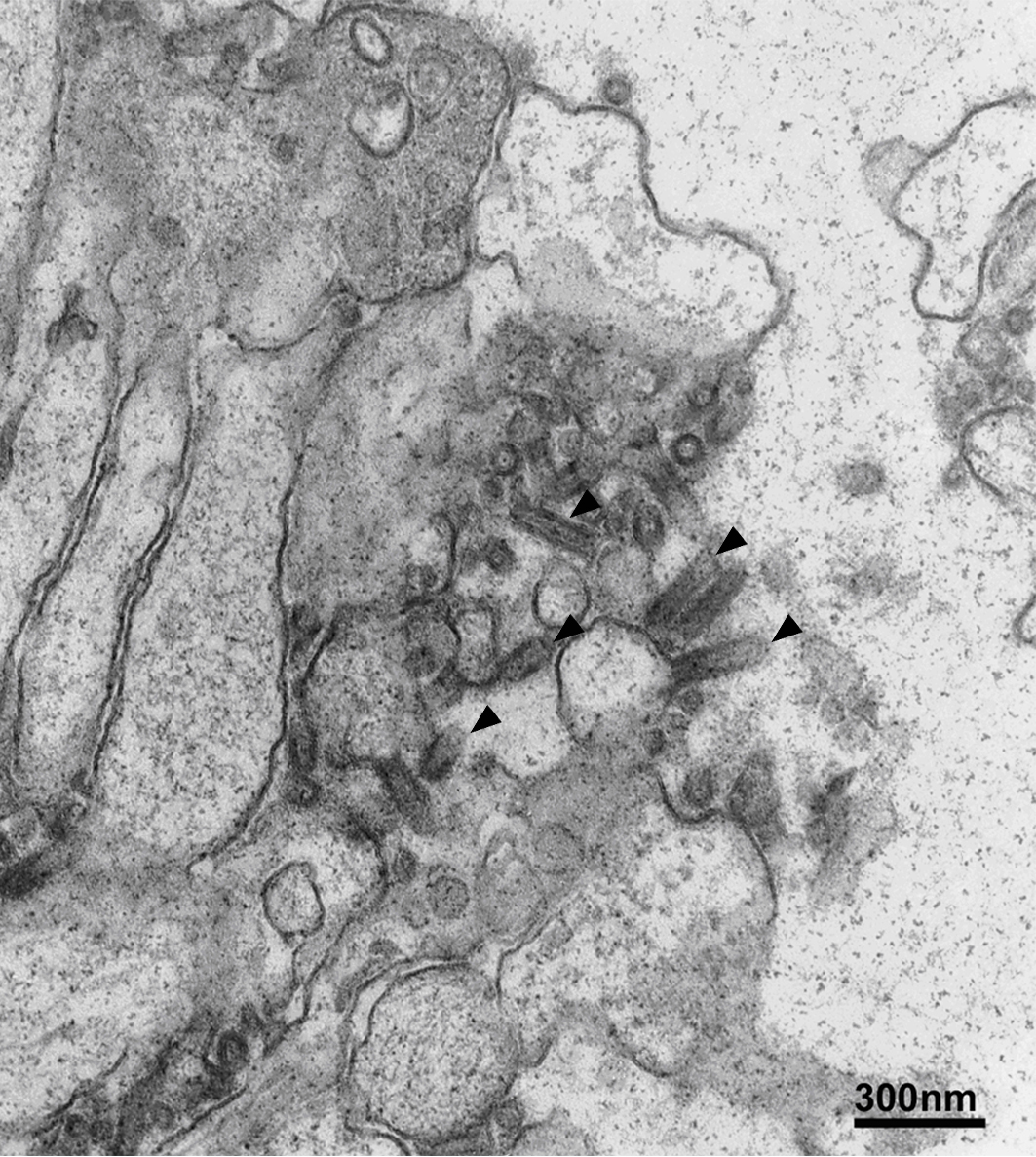

Supplement: Figure S2 — Electron micrograph of Piry virus in the cerebral cortex. Electron micrograph of Piry virus in the cerebral cortex of neonate albino Swiss mice used to prepare infected brain homogenate. Note the typical bullet morphology of this Rhabdovirus species (arrow heads). (TIF) [file pone.0015597.s003.tif]

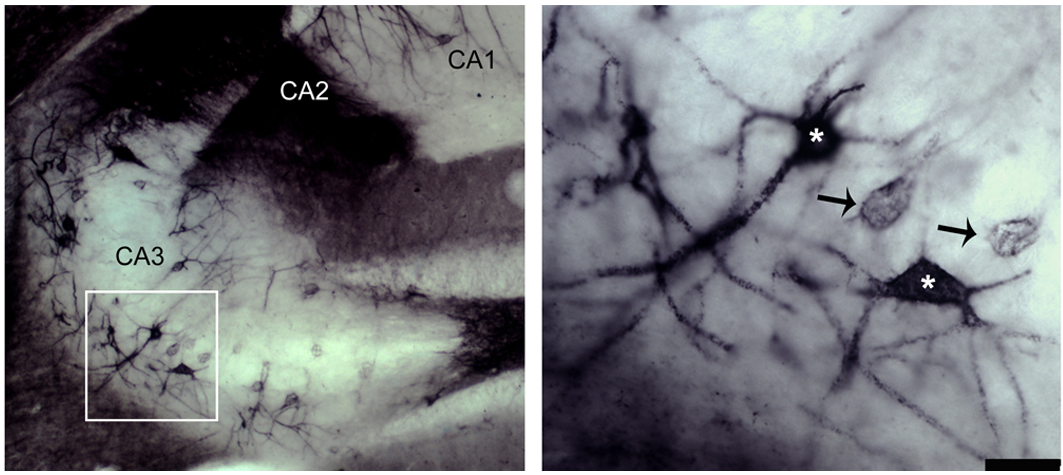

Supplement: Figure S3 — CA3 limits and perineuronal net types. Photomicrographs of histochemically reacted parasagittal sections of the architectonic limits of CA3 (low power) and types of perineuronal nets (high power). The CA3 pyramidal cell layer was outlined after histochemical reactions for Wisteria floribunda lectin (A) and immunohistochemistry for NeuN (not illustrated). Note that the Wisteria floribunda histochemical reaction labeled two types of perineuronal nets, indicated in the picture as type I and II. Arrows point to type II and asterisk to type I perineuronal nets. Scale bars: low power 250 µm; high power 25 µm. (TIF) [file pone.0015597.s004.tif]
