# Supplementary material for: Influence of Enriched Environment on Viral Encephalitis Outcomes: Behavioral and Neuropathological Changes in Albino Swiss Mice
Source: PLoS One. 2011 Jan 11;6(1):e15597. doi: 10.1371/journal.pone.0015597 (PMC3019164; doi:10.1371/journal.pone.0015597)
Supplement: Table S1 — Microglial and perineuronal net estimations at 8 d post inoculation. (DOC) [file pone.0015597.s005.doc]

Table S1. Individual unilateral microglia and perineuronal net estimations (n) with coefficients of error (CE) for CA3 in adult female albino Swiss mice 8 d post-inoculation with Piry virus-infected brain homogenates

| ***Microglia*** | | | | ***Perineuronal nets*** | | | | | |  |
| --- | --- | --- | --- | --- | --- | --- | --- | --- | --- | --- |
| ***Subjects*** | ***Section***  ***thickness*** | ***N*** | ***CE*** | ***Subjects*** | ***Section***  ***thickness*** | ***N***  ***(type I)*** | ***N***  ***(type II)*** | ***N***  ***(Total)*** | ***CE*** | |
|  |  |  |  | IEPY 2 | 19.37 ± 0.34 | 491 | 684 | 1175 | 0.093 | |
| IEPY 1 | 19.04 ± 0.41 | 34205 | 0.03 | IEPY 1 | 21.71 ± 0.85 | 462 | 656 | 1119 | 0.089 | |
| IEPY 3 | 18.35 ± 0.52 | 37355 | 0.03 | IEPY 3 | 18.3 ± 0.75 | 468 | 536 | 1004 | 0.082 | |
| IEPY 5 | 19.68 ± 0.4 | 38493 | 0.02 | IEPY 5 | 20.18 ± 0.10 | 427 | 953 | 1380 | 0.088 | |
| IEPY 15 | 20.15 ± 0.1 | 33105 | 0.03 | IEPY 15 | 20.71 ± 0.69 | 421 | 889 | 1309 | 0.086 | |
| IEPY 4 | 19.96 ± 0.14 | 30376 | 0.03 | IEPY 4 | 19.19 ± 0.46 | 566 | 831 | 1398 | 0.075 | |
| IEPY mean | 19.00 ± 0.32 | 34707 | 0.03 | IEPY mean | 20.02 ± 0.59 | 473 | 758 | 1174 | 0.084 | |
| SD |  | 3275.78 |  | S.D. |  | 53 | 158.673 | 173.31 |  | |
| CV^2^= (SD/mean)^2^ |  | 0.009 |  | CV^2^= (SD/mean)^2^ |  | 0.013 | 0.0438 | 0.022 |  | |
| CE^2^ |  | 0.0008 |  | CE^2^ |  | 0.014 | 0.01 | 0.0071 |  | |
| CE^2^/CV^2^ |  | 0.094 |  | CE^2^/CV^2^ |  | 1.08 | 0.22833 | 0.325 |  | |
| CVB^2^ |  | 0.008 |  | CVB^2^ |  | -0.001 | 0.0338 | 0.015 |  | |
| CVB^2^ (% of CV^2^) |  | 91 |  | CVB^2^ (% of CV^2^) |  | -7.69 | 77.17 | 67.54 |  | |
| EEPY 1 | 22.40 ± 0.15 | 25043 | 0.034 | EEPY 1 | 18.58 ± 0.23 | 788 | 563 | 1352 | 0.077 | |
| EEPY 2 | 19.70 ± 0.52 | 22382 | 0.029 | EEPY 2 | 19.26 ± 0.24 | 657 | 715 | 1371 | 0.079 | |
| EEPY 3 | 19.62 ± 0.41 | 15794 | 0.038 | EEPY 3 | 18.00 ± 0.17 | 798 | 729 | 1528 | 0.073 | |
| EEPY 4 | 19.30 ± 0.22 | 29533 | 0.032 | EEPY 4 | 18.25 ± 0.22 | 709 | 709 | 1418 | 0.075 | |
| EEPY 5 | 19.10 ± 0.19 | 20697 | 0.040 | EEPY 5 | 19.86 ± 0.10 | 623 | 1178 | 1800 | 0.072 | |
| EEPY mean | 20.02 ± 0.60 | 22690 | 0.034 | EEPY mean | 18.79 ± 0.34 | 715 | 779 | 1494 | 0.075 | |
| SD |  | 5099 |  | SD |  | 77.82 | 233.19 | 184.56 |  | |
| CV^2^= (SD/mean)^2^ |  | 0.050 |  | CV^2^= (SD/mean)^2^ |  | 0.0118 | 0.0896 | 0.015 |  | |
| CE^2^ |  | 0.001 |  | CE^2^ |  | 0.009 | 0.0089 | 0.006 |  | |
| CE^2^/CV^2^ |  | 0.0223 |  | CE^2^/CV^2^ |  | 0.762 | 0.0994 | 0.39 |  | |
| CVB^2^ |  | 0.049 |  | CVB^2^ |  | 0.0028 | 0.081 | 0.009 |  | |
| CVB^2^ (% of CV^2^) |  | 98 |  | CVB^2^ (% of CV^2^) |  | 23.79 | 90.058 | 60.69 |  | |

Data are given as mean group numbers (N), standard deviation (SD), and individual and mean CEs.

IEPY, impoverished environment; EEPY, enriched environment; CVB^2^ = CV^2^ – CE^2^ (CV coefficient of variation; CVB, biological coefficient of variation).
