# Supplementary material for: Influence of Enriched Environment on Viral Encephalitis Outcomes: Behavioral and Neuropathological Changes in Albino Swiss Mice
Source: PLoS One. 2011 Jan 11;6(1):e15597. doi: 10.1371/journal.pone.0015597 (PMC3019164; doi:10.1371/journal.pone.0015597)
Supplement: Table S2 — Microglial and perineuronal net estimations at 20 d post inoculation. (DOC) [file pone.0015597.s006.doc]

Table S2. Individual unilateral activated microglia and perineuronal net numbers (n) with coefficients of error (CE) for CA3 in adult female albino Swiss mice 20 d post-inoculation with Piry-virus–infected or normal brain homogenates.

| ***Microglia*** | | | | ***Perineuronal nets*** | | | | | |  |
| --- | --- | --- | --- | --- | --- | --- | --- | --- | --- | --- |
| ***Subjects*** | ***Section***  ***thickness*** | ***N*** | ***CE*** | ***Subjects*** | ***Section***  ***thickness*** | ***N***  ***(type I)*** | ***N***  ***(type II)*** | ***N***  ***(Total)*** | ***CE*** | |
| IECT 8 | 19.61 ± 0.23 | 10934 | 0.047 | IECT 8 | 21.27 ± 0.06 | 609 | 686 | 1294 | 0.080 | |
| IECT 14 | 22.45 ± 0.26 | 7078 | 0.073 | IECT 14 | 21.48 ± 0.14 | 944 | 1576 | 2521 | 0.079 | |
| IECT 15 | 19.37 ± 0.36 | 13745 | 0.045 | IECT 15 | 21.74 ± 0.08 | 643 | 1030 | 1673 | 0.093 | |
| IECT 23 | 21.88 ± 0.21 | 9593 | 0.055 | IECT 23 | 22.04 ± 0.21 | 621 | 1275 | 1897 | 0.076 | |
| IECT mean |  | 10337 | 0.055 | IECT mean | 21.63 ± 0.17 | 704 | 1142 | 1846 | 0.082 | |
| SD |  | 2777.82 |  | SD |  | 160.73 | 377.41 | 513.82 |  | |
| CV^2^= (SD/mean)^2^ |  | 0.072206 |  | CV^2^= (SD/mean)^2^ |  | 0.052 | 0.109 | 0.077 |  | |
| CE^2^ |  | 0.003025 |  | CE^2^ |  | 0.011 | 0.0099 | 0.007 |  | |
| CE^2^/CV^2^ |  | 0.041894 |  | CE^2^/CV^2^ |  | 0.207 | 0.091 | 0.09 |  | |
| CVB^2^ |  | 0.069181 |  | CVB^2^ |  | 0.0413 | 0.099 | 0.07 |  | |
| CVB^2^ (% of CV^2^) |  | 95.81062 |  | CVB^2^ (% of CV^2^) |  | 79.28 | 90.9 | 90.96 |  | |
| IEPY 1 | 19.60 ± 0.19 | 16481 | 0.038 | IEPY 9 | 21.30 ± 0.13 | 465 | 1285 | 1750 | 0.074 | |
| IEPY 4 | 19.03 ± 0.11 | 15323 | 0.047 | IEPY 4 | 21.30 ± 0.12 | 350 | 1192 | 1542 | 0.087 | |
| IEPY 12 | 21.15 ± 0.08 | 7647 | 0.072 | IEPY 12 | 21.70 ± 0.15 | 411 | 1273 | 1684 | 0.081 | |
| IEPY 13 | 20.06 ± 0.24 | 17685 | 0.036 | IEPY 13 | 20.86 ± 0.03 | 402 | 728 | 1130 | 0.079 | |
| IEPY 19 | 20.17 ± 0.08 | 20053 | 0.039 | IEPY 19 | 21.50 ± 0.14 | 300 | 1194 | 1494 | 0.081 | |
| IEPY 20 | 18.74 ± 0.07 | 14913 | 0.040 | IEPY 20 | 22.17 ± 0.23 | 861 | 828 | 1689 | 0.071 | |
| IEPY mean | 19.50 ± 0.36 | 15350 | 0.040 | IEPY mean | 21.34 ± 0.18 | 465 | 1083 | 1520 | 0.079 | |
| SD |  | 4204.45 |  | S.D. |  | 202.29 | 241.73 | 226.64 |  | |
| CV^2^= (SD/mean)^2^ |  | 0.075 |  | CV^2^= (SD/mean)^2^ |  | 0.18934 | 0.04978 | 0.022 |  | |
| CE^2^ |  | 0.002 |  | CE^2^ |  | 0.01517 | 0.00809 | 0.0062 |  | |
| CE^2^/CV^2^ |  | 0.027336 |  | CE^2^/CV^2^ |  | 0.08012 | 0.16243 | 0.2795114 |  | |
| CVB^2^ |  | 0.073 |  | CVB^2^ |  | 0.17417 | 0.0417 | 0.016 |  | |
| CVB^2^ (% of CV^2^) |  | 97.27 |  | CVB^2^ (% of CV^2^) |  | 91.99 | 83.76 | 72.01 |  | |
| EECT 7 | 18.72 ± 0.11 | 13930 | 0.043 | EECT 7 | 22.17 ± 0.17 | 1041 | 635 | 1676 | 0.074 | |
| EECT 23 | 18.49 ± 0.06 | 9069 | 0.062 | EECT 23 | 22.09 ± 0.09 | 953 | 1269 | 1444 | 0.080 | |
| EECT 15 | 20.78 ± 0.16 | 13489 | 0.043 | EECT 15 | 22.04 ± 0.12 | 841 | 603 | 2222 | 0.078 | |
| EECT 25 | 20.93 ± 0.11 | 11459 | 0.053 | EECT 25 | 21.29 ± 0.09 | 747 | 1041 | 1789 | 0.065 | |
| IECT mean | 19.45 ± 1.30 | 11987 | 0.050 | IECT mean | 21.90 ± 0.20 | 896 | 887 | 1781 | 0.074 | |
| SD |  | 2222.97 |  | SD |  | 128.32 | 323.18 | 325.95 |  | |
| CV^2^= (SD/mean)^2^ |  | 0.034391 |  | CV^2^= (SD/mean)^2^ |  | 0.02 | 0.1327 | 0.034 |  | |
| CE^2^ |  | 0.002525 |  | CE^2^ |  | 0.008 | 0.010 | 0.006 |  | |
| CE^2^/CV^2^ |  | 0.073421 |  | CE^2^/CV^2^ |  | 0.388 | 0.0786 | 0.165 |  | |
| CVB^2^ |  | 0.031866 |  | CVB^2^ |  | 0.0126 | 0.122 | 0.028 |  | |
| CVB^2^ (% of CV^2^) |  | 92.65786 |  | CVB^2^ (% of CV^2^) |  | 61.2 | 92.13 | 83.5 |  | |
| EEPY 2 | 18.91 ± 0.14 | 6129 | 0.078 | EEPY 19 | 22.02 ± 0.18 | 841 | 603 | 1444 | 0.08 | |
| EEPY 10 | 18.47 ± 0.04 | 6387 | 0.075 | EEPY 10 | 20.58 ± 0.17 | 770 | 851 | 1620 | 0.077 | |
| EEPY 18 | 18.47 ± 0.10 | 9128 | 0.062 | EEPY 18 | 22.00 ± 0.14 | 808 | 1116 | 1925 | 0.075 | |
| EEPY 21 | 18.44 ± 0.06 | 14295 | 0.043 | EEPY 21 | 22.04 ± 0.30 | 222 | 699 | 920 | 0.10 | |
| EEPY 22 | 18.63 ± 0.10 | 6071 | 0.08 | EEPY 22 | 22.00 ± 0.07 | 1158 | 1305 | 2463 | 0.081 | |
| EEPY mean | 18.58 ± 0.09 | 8402 | 0.068 | EEPY mean | 21.73 ± 0.29 | 894 | 291.81 | 1674 | 0.083 | |
| SD |  | 3532.56 |  | SD |  | 338.04 | 0.09073 | 571.92 |  | |
| CV^2^= (SD/mean)^2^ |  | 0.177 |  | CV^2^= (SD/mean)^2^ |  | 0.14289 | 0.09073 | 0.117 |  | |
| CE^2^ |  | 0.00457 |  | CE^2^ |  | 0.00925 | 0.01066 | 0.007 |  | |
| CE^2^/CV^2^ |  | 0.025853 |  | CE^2^/CV^2^ |  | 0.06472 | 0.11748 | 0.060 |  | |
| CVB^2^ |  | 0.172 |  | CVB^2^ |  | 0.13364 | 0.0801 | 0.11 |  | |
| CVB^2^ (% of CV^2^) |  | 97.41 |  | CVB^2^ (% of CV^2^) |  | 93.53 | 88.25 | 94 |  | |

The data are presented as mean group numbers (N), standard deviation (SD), and individual and mean CEs.

EECT, enriched environment control; IECT, impoverished environment control; EEPY, enriched environment. Piry infected; IEPY, impoverished environment. Piry infected; CVB^2^ = CV^2^ – CE^2^ (CV coefficient of variation; CVB, biological coefficient of variation).
