# Supplementary material for: Influence of Enriched Environment on Viral Encephalitis Outcomes: Behavioral and Neuropathological Changes in Albino Swiss Mice
Source: PLoS One. 2011 Jan 11;6(1):e15597. doi: 10.1371/journal.pone.0015597 (PMC3019164; doi:10.1371/journal.pone.0015597)
Supplement: Table S3 — Microglial and perineuronal net estimations at 40 d post inoculation. (DOC) [file pone.0015597.s007.doc]

Table S3. Individual unilateral microglia and perineuronal net estimations (n) with coefficients of error (CE) for CA3 in adult female albino Swiss mice 40 d post-inoculation with Piry-virus–infected brain homogenate.

| ***Microglia*** | | | | ***Perineuronal nets*** | | | | | |  |
| --- | --- | --- | --- | --- | --- | --- | --- | --- | --- | --- |
| ***Subjects*** | ***Section***  ***thickness*** | ***N*** | ***CE*** | ***Subjects*** | ***Section***  ***thickness*** | ***N***  ***(type I)*** | ***N***  ***(type II)*** | ***N***  ***(Total)*** | ***CE*** | |
| IEPY 2 | 18.83 ± 0.11 | 19128 | 0.038 | IEPY 2 | 17.28 ± 0.1 | 531 | 699 | 1229 | 0.077 | |
| IEPY 6 | 22.23 ± 1.01 | 13613 | 0.056 | IEPY 6 | 16.96 ± 0.08 | 552 | 794 | 1346 | 0.064 | |
| IEPY 7 | 18.71 ± 0.07 | 24789 | 0.035 | IEPY 7 | 17.2 ± 0.15 | 948 | 1104 | 2051 | 0.064 | |
| IEPY 12 | 18.62 ± 0.17 | 25413 | 0.034 | IEPY 12 | 18.81 ± 0.43 | 759 | 1046 | 1805 | 0.074 | |
| IEPY 13 | 20.1 ± 0.13 | 12477 | 0.054 | IEPY 13 | 17.29 ± 0.18 | 512 | 700 | 1213 | 0.074 | |
| IEPY mean | 19.2 ± 0.8 | 19084 | 0.043 | IEPY mean |  | 660 | 869 | 1529 |  | |
| SD |  | 6045.44 |  | SD |  | 188.99 | 193.43 | 378.33 |  | |
| CV^2^= (SD/mean)^2^ |  | 0.1 |  | CV^2^= (SD/mean)^2^ |  | 0.08187 | 0.04958 | 0.061 |  | |
| CE^2^ |  | 0.002 |  | CE^2^ |  | 0.00848 | 0.008 | 0.005 |  | |
| CE^2^/CV^2^ |  | 0.016454 |  | CE^2^/CV^2^ |  | 0.10352 | 0.16135 | 0.082 |  | |
| CVB^2^ |  | 0.099 |  | CVB^2^ |  | 0.0734 | 0.04158 | 0.056 |  | |
| CVB^2^ (% of CV^2^) |  | 98.35 |  | CVB^2^ (% of CV^2^) |  | 89.65 | 83.86 | 91.8 |  | |
|  |  |  |  | EEPY 8 | 17.63 ± 0.15 | 790 | 843 | 1632 | 0.067 | |
| EEPY 3 | 18.98 ± 0.032 | 7042 | 0.067 | EEPY 3 | 17.6 ± 0.22 | 965 | 742 | 1707 | 0.072 | |
| EEPY 11 | 18.93 ± 0.095 | 13018 | 0.050 | EEPY 11 | 18.73 ± 0.42 | 1206 | 985 | 2191 | 0.064 | |
| EEPY 16 | 20.03 ± 0.31 | 13912 | 0.042 | EEPY 16 | 17.6 ± 0.3 | 479 | 459 | 939 | 0.094 | |
| EEPY 18 | 21.22 ± 1.35 | 14000 | 0.046 | EEPY 18 | 17.73 ± 0.32 | 1168 | 920 | 2089 | 0.06 | |
| EEPY mean | 19.25 ± 0.075 | 11993 | 0.051 | EEPY mean |  | 921 | 790 | 1711 |  | |
| SD |  | 3330.36 |  | SD |  | 298.34 | 205.91 | 493.91 |  | |
| CV^2^= (SD/mean)^2^ |  | 0.077114 |  | CV^2^= (SD/mean)^2^ |  | 0.1048 | 0.06794 | 0.08328 |  | |
| CE^2^ |  | 0.003 |  | CE^2^ |  | 0.00798 | 0.00876 | 0.005 |  | |
| CE^2^/CV^2^ |  | 0.034115 |  | CE^2^/CV^2^ |  | 0.07617 | 0.12893 | 0.06 |  | |
| CVB^2^ |  | 0.074 |  | CVB^2^ |  | 0.09683 | 0.05918 | 0.078 |  | |
| CVB^2^ (% of CV^2^) |  | 96.59 |  | CVB^2^ (% of CV^2^) |  | 92.38 | 87.11 | 94 |  | |

The data are given as mean group numbers (N), standard deviation (SD), and individual and mean CEs.

EEPY, enriched environment; IEPY, impoverished environment; CVB^2^ = CV^2^ – CE^2^ (CV coefficient of variation; CVB, biological coefficient of variation).
