# Supplementary material for: Influence of Enriched Environment on Viral Encephalitis Outcomes: Behavioral and Neuropathological Changes in Albino Swiss Mice
Source: PLoS One. 2011 Jan 11;6(1):e15597. doi: 10.1371/journal.pone.0015597 (PMC3019164; doi:10.1371/journal.pone.0015597)
Supplement: Table S4 — Neuronal estimations at 20 d post inoculation. (DOC) [file pone.0015597.s008.doc]

Table S4. Individual unilateral neuron numbers (n) with coefficients of error (CE) for CA3 in adult female albino Swiss mice 20 d after nasal instillation of Piry (PY)-infected or normal brain homogenates (contr.)

| ***Neurons*** | | | | | | | | |
| --- | --- | --- | --- | --- | --- | --- | --- | --- |
| ***Subjects*** | ***Section***  ***thickness*** | ***N*** | ***CE*** | ***Subjects*** | ***Section***  ***thickness*** | ***N*** | ***CE*** |  |
| IECont 7 | 17.29 ± 0.22 | 33374 | 0.045 | EECont 6 | 17.41 ± 0.44 | 38998 | 0.046 |  |
| IECont 8 | 17.37 ± 0.22 | 36319 | 0.050 | EECont 7 | 22.18 ± 0.12 | 36483 | 0.046 |  |
| IECont 15 | 17.09 ± 0.10 | 37963 | 0.048 | EECont 23 | 20.82 ± 0.16 | 30969 | 0.055 |  |
| IECont 23 | 22.19 ± 0.19 | 35311 | 0.055 | EECont 25 | 22.08 ± 0.11 | 33967 | 0.054 |  |
| IECont mean | 18.48 ± 1.24 | 35742 | 0.050 | EECont mean | 20.62 ± 1.11 | 35104 | 0.050 |  |
| SD |  | 1919.87 |  | SD |  | 3437.89 |  |  |
| CV^2^= (SD/mean)^2^ |  | 0.002885 |  | CV^2^= (SD/mean)^2^ |  | 0.00959 |  |  |
| CE^2^ |  | 0.00245 |  | CE^2^ |  | 0.00252 |  |  |
| CE^2^/CV^2^ |  | 0.849228 |  | CE^2^/CV^2^ |  | 0.26327 |  |  |
| CVB^2^ |  | 0.000435 |  | CVB^2^ |  | 0.00707 |  |  |
| CVB^2^ (% of CV^2^) |  | 15.08 |  | CVB^2^ (% of CV^2^) |  | 73.67 |  |  |
| IEPY 1 | 17.31 ± 0.40 | 49670 | 0.040 | EEPY 10 | 17.05 ± 0.24 | 36466 | 0.045 |  |
| IEPY 2 | 17.18 ± 0.42 | 35005 | 0.052 | EEPY 18 | 17.10 ± 0.18 | 29781 | 0.046 |  |
| IEPY 13 | 24.92 ± 0.12 | 30661 | 0.059 | EEPY 21 | 20.97 ± 0.06 | 29023 | 0.057 |  |
| IEPY 20 | 17.04 ± 0.19 | 33641 | 0.050 | EEPY 22 | 17.01 ± 0.60 | 38714 | 0.046 |  |
| IEPY mean | 18.92 ± 2.00 | 37244 | 0.050 | EEPY mean | 18.03 ± 0.98 | 33496 | 0.048 |  |
| SD |  | 8480.01 |  | SD |  | 4825.42 |  |  |
| CV^2^= (SD/mean)^2^ |  | 0.051842 |  | CV^2^= (SD/mean)^2^ |  | 0.02075 |  |  |
| CE^2^ |  | 0.002525 |  | CE^2^ |  | 0.00235 |  |  |
| CE^2^/CV^2^ |  | 0.048707 |  | CE^2^/CV^2^ |  | 0.11334 |  |  |
| CVB^2^ |  | 0.049317 |  | CVB^2^ |  | 0.01840 |  |  |
| CVB^2^ (% of CV^2^) |  | 95.13 |  | CVB^2^ (% of CV^2^) |  | 88.67 |  |  |

The data are given as mean group numbers (N), standard deviation (SD), and individual and mean CEs. EE, enriched environment; IE, impoverished environment; CVB^2^ = CV^2^ – CE^2^ (CV coefficient of variation; CVB, biological coefficient of variation).
