# Supplementary material for: Influence of Enriched Environment on Viral Encephalitis Outcomes: Behavioral and Neuropathological Changes in Albino Swiss Mice
Source: PLoS One. 2011 Jan 11;6(1):e15597. doi: 10.1371/journal.pone.0015597 (PMC3019164; doi:10.1371/journal.pone.0015597)
Supplement: Table S5 — Correlations between microglial activation and extracellular matrix damage. (DOC) [file pone.0015597.s009.doc]

Table S5. Linear regression results illustrating an inverse correlation between the degree of microglia activation and perineuronal net (PN) reduction

| **Microglia x total PNs** | **8 dpi** | **20 dpi** | **40 dpi** |
| --- | --- | --- | --- |
| F | 14.2690 | 6.1741 | 0.0256 |
| *p* | 0.0019* | 0.0249 | 0.8692 |
| R^2^ | 0.4384 | 0.2565 | -0.0648 |
| Coefficient of correlation | 0.6868^#^ | 0.5532 | 0.0413 |
| **Microglia x type I PNs** |  |  |  |
| F | 17.3633 | 9.3293 | 0.0408 |
| *p* | 0.0010* | 0.0084* | 0.8365 |
| R^2^ | 0.4905 | 0.3570 | -0.0638 |
| Coefficient of correlation | 0.7214^#^ | 0.6324^#^ | 0.0521 |
| **Microglia x type II PNs** |  |  |  |
| F | 3.6033 | 0.8062 | 0.0053 |
| *p* | 0.0729 | 0.6120 | 0.9411 |
| R^2^ | 0.1328 | -0.0131 | -0.0663 |
| Coefficient of correlation | 0.4287 | 0.2333 | 0.0188 |

NNote that type I PNs presented higher coefficients of correlation with microglial estimations than type II or total PN estimations. dpi, days post-inoculation.

(*) indicates significant *p* level, and (^#^) coefficient of correlation > 60%.
