# Supplementary material for: Influence of Enriched Environment on Viral Encephalitis Outcomes: Behavioral and Neuropathological Changes in Albino Swiss Mice
Source: PLoS One. 2011 Jan 11;6(1):e15597. doi: 10.1371/journal.pone.0015597 (PMC3019164; doi:10.1371/journal.pone.0015597)
Supplement: Table S6 — Stereological parameters for microglial estimations and counted markers. (DOC) [file pone.0015597.s010.doc]

Table S6. Experimental parameters and counting results from the optical fractionator(1): activated microglial counted markers (∑q-) of CA3 in adult female albino Swiss mice at 8, 20, and 40 d post-nasal instillation with Piry-virus–infected or normal brain homogenates (1)

| *Subjects* | *a(frame)*  *(µm^2^)* | *A(x,y step)*  *(µm^2^)* | *asf* | *tsf* | *ssf* | *No. of counting frames* | *No. of sections* | *ΣQ^-^* |
| --- | --- | --- | --- | --- | --- | --- | --- | --- |
| IEPY 1 (8 dpi) | 40 x 40 | 80 x 80 | 0.25 | 0.497 ± 0.0112 | 1/6 | 240 | 5 | 748 |
| IEPY 3 (8 dpi) | 40 x 40 | 80 x 80 | 0.25 | 0.548 ± 0.0167 | 1/6 | 251 | 5 | 845 |
| IEPY 4 (8 dpi) | 40 x 40 | 80 x 80 | 0.25 | 0.502 ± 0.0035 | 1/6 | 260 | 5 | 635 |
| IEPY 5 (8 dpi) | 40 x 40 | 80 x 80 | 0.25 | 0.511 ± 0.0108 | 1/6 | 273 | 5 | 811 |
| IEPY 15 (8 dpi) | 40 x 40 | 80 x 80 | 0.25 | 0.497 ± 0.0025 | 1/6 | 339 | 5 | 682 |
| EEPY 1 (8 dpi) | 40 x 40 | 80 x 80 | 0.25 | 0.448 ± 0.0030 | 1/6 | 251 | 5 | 464 |
| EEPY 2 (8 dpi) | 40 x 40 | 80 x 80 | 0.25 | 0.512 ± 0.0130 | 1/6 | 213 | 4 | 471 |
| EEPY 3 (8 dpi) | 40 x 40 | 80 x 80 | 0.25 | 0.511 ± 0.0111 | 1/6 | 232 | 5 | 331 |
| EEPY 4 (8 dpi) | 40 x 40 | 80 x 80 | 0.25 | 0.520 ± 0.0057 | 1/6 | 211 | 5 | 633 |
| EEPY 5 (8 dpi) | 40 x 40 | 80 x 80 | 0.25 | 0.527 ± 0.0051 | 1/6 | 271 | 5 | 451 |
| IEcont 8 (20 dpi) | 40 x 40 | 80 x 80 | 0.25 | 0.511 ± 0.0068 | 1/6 | 189 | 5 | 234 |
| IEcont 14 (20dpi) | 40 x 40 | 80 x 80 | 0.25 | 0.445 ± 0.0052 | 1/6 | 222 | 5 | 132 |
| IEcont 15 (20dpi) | 40 x 40 | 80 x 80 | 0.25 | 0.518 ± 0.0093 | 1/6 | 198 | 5 | 297 |
| IEcont 23 (20dpi) | 40 x 40 | 80 x 80 | 0.25 | 0.458 ± 0.0044 | 1/6 | 207 | 6 | 181 |
| IEPY 1 (20dpi) | 40 x 40 | 80 x 80 | 0.25 | 0.511 ± 0.0049 | 1/6 | 222 | 5 | 350 |
| IEPY 4 (20dpi) | 40 x 40 | 80 x 80 | 0.25 | 0.526 ± 0.0030 | 1/6 | 244 | 5 | 334 |
| IEPY 12 (20dpi) | 40 x 40 | 80 x 80 | 0.25 | 0.473 ± 0.0019 | 1/6 | 236 | 6 | 151 |
| IEPY 13 (20dpi) | 40 x 40 | 80 x 80 | 0.25 | 0.500 ± 0.0060 | 1/6 | 221 | 5 | 366 |
| IEPY 19 (20dpi) | 40 x 40 | 80 x 80 | 0.25 | 0.496 ± 0.0020 | 1/6 | 262 | 5 | 412 |
| IEPY 20 (20dpi) | 40 x 40 | 80 x 80 | 0.25 | 0.534 ± 0.0020 | 1/6 | 236 | 5 | 332 |
| EEcont 7 (20dpi) | 40 x 40 | 80 x 80 | 0.25 | 0.535 ± 0.0033 | 1/6 | 287 | 6 | 312 |
| EEcont 15 (20dpi) | 40 x 40 | 80 x 80 | 0.25 | 0.482 ± 0.0038 | 1/6 | 283 | 6 | 273 |
| EEcont 23 (20dpi) | 40 x 40 | 80 x 80 | 0.25 | 0.541 ± 0.0019 | 1/6 | 217 | 5 | 205 |
| EEcont 25 (20dpi) | 40 x 40 | 80 x 80 | 0.25 | 0.477 ± 0.0030 | 1/6 | 306 | 5 | 228 |
| EEPY 2 (20dpi) | 40 x 40 | 80 x 80 | 0.25 | 0.530 ± 0.0042 | 1/6 | 242 | 5 | 134 |
| EEPY 10 (20dpi) | 40 x 40 | 80 x 80 | 0.25 | 0.542 ± 0.0011 | 1/6 | 198 | 5 | 144 |
| EEPY 18 (20dpi) | 40 x 40 | 80 x 80 | 0.25 | 0.542 ± 0.0019 | 1/6 | 252 | 5 | 205 |
| EEPY 21 (20dpi) | 40 x 40 | 80 x 80 | 0.25 | 0.543 ± 0.0018 | 1/6 | 291 | 5 | 324 |
| EEPY 22 (20dpi) | 40 x 40 | 80 x 80 | 0.25 | 0.537 ± 0.0030 | 1/6 | 207 | 5 | 136 |
| IEPY 2 (40dpi) | 40 x 40 | 80 x 80 | 0.25 | 0.532 ± 0.0031 | 1/6 | 297 | 6 | 426 |
| IEPY 6 (40dpi) | 40 x 40 | 80 x 80 | 0.25 | 0.456 ± 0.0208 | 1/6 | 272 | 6 | 260 |
| IEPY 7 (40dpi) | 40 x 40 | 80 x 80 | 0.25 | 0.535 ± 0.0021 | 1/6 | 303 | 5 | 554 |
| IEPY 12 (40dpi) | 40 x 40 | 80 x 80 | 0.25 | 0.538 ± 0.0050 | 1/6 | 342 | 6 | 563 |
| IEPY 13 (40dpi) | 40 x 40 | 80 x 80 | 0.25 | 0.498 ± 0.0032 | 1/6 | 309 | 6 | 259 |
| EEPY 3 (40dpi) | 40 x 40 | 80 x 80 | 0.25 | 0.527 ± 0.0009 | 1/6 | 225 | 6 | 154 |
| EEPY 11 (40dpi) | 40 x 40 | 80 x 80 | 0.25 | 0.529 ± 0.0026 | 1/6 | 302 | 6 | 284 |
| EEPY 16 (40dpi) | 40 x 40 | 80 x 80 | 0.25 | 0.501 ± 0.0075 | 1/6 | 258 | 6 | 291 |
| EEPY 18 (40dpi) | 40 x 40 | 80 x 80 | 0.25 | 0.481 ± 0.0293 | 1/6 | 225 | 5 | 263 |

^1)^Area of the optical dissector counting frame, a(frame); x and y step sizes, A(x,y step); asf, area sampling fraction [a(frame)/A(x,y step)]; tsf, thickness sampling fraction, calculated by the height of the optical dissector divided by section thickness, h/section thickness. ssf, section sampling fraction.

^2)^ All evaluations were performed with a 60X objective lens (N.A. 1.4; D.F. 0.75 µm).
