# Supplementary material for: Influence of Enriched Environment on Viral Encephalitis Outcomes: Behavioral and Neuropathological Changes in Albino Swiss Mice
Source: PLoS One. 2011 Jan 11;6(1):e15597. doi: 10.1371/journal.pone.0015597 (PMC3019164; doi:10.1371/journal.pone.0015597)
Supplement: Table S7 — Stereological parameters for perineuronal net estimations and counted markers. (DOC) [file pone.0015597.s011.doc]

Table S7. Experimental parameters and counting results from the optical fractionator(1): perineuronal net counted markers (∑q-) of CA3 in adult female albino Swiss mice at days 8, 20, and 40 d post-nasal instillation with Piry-virus–infected or normal brain homogenates (1)

| *Animais* | *a(frame)*  *(µm^2^)* | *A(x,y step)*  *(µm^2^)* | *asf* | *tsf* | *ssf* | *No. of counting frames* | *No. of sections* | *ΣQ^-^* |
| --- | --- | --- | --- | --- | --- | --- | --- | --- |
| IEPY 1 (8 dpi) | 120x120 | 120x120 | 1 | 0.752 ± 0.0300 | 1/6 | 125 | 5 | 133 |
| IEPY 2 (8 dpi) | 120x120 | 120x120 | 1 | 0.832 ± 0.0140 | 1/6 | 132 | 6 | 158 |
| IEPY 3 (8 dpi) | 120x120 | 120x120 | 1 | 0.884 ± 0.0300 | 1/6 | 129 | 6 | 148 |
| IEPY 4 (8 dpi) | 120x120 | 120x120 | 1 | 0.841 ± 0.0200 | 1/6 | 166 | 6 | 190 |
| IEPY 5 (8 dpi) | 120x120 | 120x120 | 1 | 0.794 ± 0.0041 | 1/6 | 111 | 6 | 181 |
| IEPY 15 (8 dpi) | 120x120 | 120x120 | 1 | 0.774 ± 0.0026 | 1/6 | 123 | 6 | 168 |
| EEPY 1 (8 dpi) | 120x120 | 120x120 | 1 | 0.866 ± 0.0090 | 1/6 | 161 | 7 | 192 |
| EEPY 2 (8 dpi) | 120x120 | 120x120 | 1 | 0.837 ± 0.0095 | 1/6 | 171 | 6 | 190 |
| EEPY 3 (8 dpi) | 120x120 | 120x120 | 1 | 0.893 ± 0.0085 | 1/6 | 177 | 7 | 222 |
| EEPY 4 (8 dpi) | 120x120 | 120x120 | 1 | 0.882 ± 0.0090 | 1/6 | 159 | 6 | 206 |
| EEPY 5 (8 dpi) | 120x120 | 120x120 | 1 | 0.806 ± 0.0039 | 1/6 | 162 | 6 | 157 |
| IEcont 8 (20dpi) | 120x120 | 120x120 | 1 | 0.942 ± 0.0026 | 1/6 | 127 | 6 | 202 |
| IEcont 14 (20dpi) | 120x120 | 120x120 | 1 | 0.933 ± 0.0058 | 1/6 | 238 | 6 | 387 |
| IEcont 15 (20dpi) | 120x120 | 120x120 | 1 | 0.922 ± 0.0032 | 1/6 | 203 | 6 | 255 |
| IEcont 23 (20dpi) | 120x120 | 120x120 | 1 | 0.910 ± 0.0085 | 1/6 | 139 | 5 | 287 |
| IEPY 4 (20dpi) | 120x120 | 120x120 | 1 | 0.940 ± 0.0052 | 1/6 | 128 | 7 | 238 |
| IEPY 9 (20dpi) | 120x120 | 120x120 | 1 | 0.939 ± 0.0055 | 1/6 | 168 | 7 | 271 |
| IEPY 12 (20dpi) | 120x120 | 120x120 | 1 | 0.924 ± 0.0062 | 1/6 | 126 | 6 | 254 |
| IEPY 13 (20dpi) | 120x120 | 120x120 | 1 | 0.960 ± 0.0014 | 1/6 | 133 | 7 | 180 |
| IEPY 19 (20dpi) | 120x120 | 120x120 | 1 | 0.934 ± 0.0058 | 1/6 | 128 | 6 | 229 |
| IEPY 20 (20dpi) | 120x120 | 120x120 | 1 | 0.904 ± 0.0090 | 1/6 | 130 | 7 | 253 |
| EEcont 7 (20dpi) | 120x120 | 120x120 | 1 | 0.905 ± 0.0067 | 1/6 | 136 | 6 | 248 |
| EEcont 15 (20dpi) | 120x120 | 120x120 | 1 | 0.910 ± 0.0047 | 1/6 | 158 | 7 | 331 |
| EEcont 23 (20dpi) | 120x120 | 120x120 | 1 | 0.910 ± 0.0155 | 1/6 | 140 | 6 | 213 |
| EEcont 25 (20dpi) | 120x120 | 120x120 | 1 | 0.940 ± 0.0039 | 1/6 | 158 | 6 | 280 |
| EEPY 10 (20dpi) | 120x120 | 120x120 | 1 | 0.976 ± 0.0082 | 1/6 | 174 | 5 | 261 |
| EEPY 18 (20dpi) | 120x120 | 120x120 | 1 | 0.911 ± 0.0055 | 1/6 | 178 | 5 | 288 |
| EEPY 19 (20dpi) | 120x120 | 120x120 | 1 | 0.912 ± 0.0071 | 1/6 | 140 | 6 | 213 |
| EEPY 21 (20dpi) | 120x120 | 120x120 | 1 | 0.911 ± 0.0114 | 1/6 | 97 | 5 | 137 |
| EEPY 22 (20dpi) | 120x120 | 120x120 | 1 | 0.916 ± 0.0027 | 1/6 | 172 | 6 | 368 |
| IEPY 2 (40dpi) | 120x120 | 120x120 | 1 | 0.927 ± 0.0052 | 1/6 | 115 | 6 | 190 |
| IEPY 6 (40dpi) | 120x120 | 120x120 | 1 | 0.944 ± 0.0042 | 1/6 | 136 | 6 | 212 |
| IEPY 7 (40dpi) | 120x120 | 120x120 | 1 | 0.932 ± 0.0080 | 1/6 | 177 | 7 | 316 |
| IEPY 12 (40dpi) | 120x120 | 120x120 | 1 | 0.857 ± 0.0194 | 1/6 | 138 | 6 | 245 |
| IEPY 13 (40dpi) | 120x120 | 120x120 | 1 | 0.927 ± 0.0091 | 1/6 | 136 | 6 | 187 |
| EEPY 3 (40dpi) | 120x120 | 120x120 | 1 | 0.912 ± 0.0109 | 1/6 | 175 | 7 | 260 |
| EEPY 8 (40dpi) | 120x120 | 120x120 | 1 | 0.910 ± 0.0076 | 1/6 | 178 | 6 | 248 |
| EEPY 11 (40dpi) | 120x120 | 120x120 | 1 | 0.856 ± 0.0186 | 1/6 | 198 | 6 | 318 |
| EEPY 16 (40dpi) | 120x120 | 120x120 | 1 | 0.912 ± 0.0146 | 1/6 | 87 | 5 | 141 |
| EEPY 18 (40dpi) | 120x120 | 120x120 | 1 | 0.908 ± 0.0151 | 1/6 | 233 | 7 | 313 |

^1)^Area of the optical dissector counting frame, a(frame); x and y step sizes, A(x,y step); asf, area sampling fraction [a(frame)/A(x,y step)]; tsf, thickness sampling fraction, calculated by the height of the optical dissector divided by section thickness, h/section thickness. ssf, section sampling fraction.

^2)^ All evaluations were performed with a 60X objective lens (N.A. 1.4; D.F. 0.75 µm).
