# Supplementary material for: Influence of Enriched Environment on Viral Encephalitis Outcomes: Behavioral and Neuropathological Changes in Albino Swiss Mice
Source: PLoS One. 2011 Jan 11;6(1):e15597. doi: 10.1371/journal.pone.0015597 (PMC3019164; doi:10.1371/journal.pone.0015597)
Supplement: Table S8 — Stereological parameters for neuronal estimations and counted markers. (DOC) [file pone.0015597.s012.doc]

Table S8. Experimental parameters and counting results from the optical fractionator^(1)^: neuronal counted markers (∑q^-^) of CA3 in adult female albino Swiss mice at 20 d post-nasal instillation with Piry-virus–infected or normal brain homogenates ^(1)^

| ***Animais*** | ***a(caixa)***  ***(µm^2^)*** | ***A(X,Y)***  ***(µm^2^)*** | ***asf*** | ***tsf*** | ***ssf*** | ***No de Caixas*** | ***No de Secções*** | ***ΣQ^-^*** |
| --- | --- | --- | --- | --- | --- | --- | --- | --- |
| IEcont 7 (20 dpi) | 30 x 30 | 60 x 60 | 0.25 | 0.417 ± 0.0046 | 1/6 | 232 | 7 | 614 |
| IEcont 8 (20dpi) | 30 x 30 | 60 x 60 | 0.25 | 0.406 ± 0.0054 | 1/6 | 190 | 7 | 617 |
| IEcont 15 (20dpi) | 30 x 30 | 60 x 60 | 0.25 | 0.415 ± 0.0025 | 1/6 | 239 | 7 | 674 |
| IEcont 23 (20dpi) | 30 x 30 | 60 x 60 | 0.25 | 0.316 ± 0.0027 | 1/6 | 186 | 6 | 464 |
| IEPY 1 (20dpi) | 30 x 30 | 60 x 60 | 0.25 | 0.436 ± 0.0128 | 1/6 | 314 | 5 | 905 |
| IEPY 2 (20dpi) | 30 x 30 | 60 x 60 | 0.25 | 0.413 ± 0.0107 | 1/6 | 215 | 7 | 596 |
| IEPY 13 (20dpi) | 30 x 30 | 60 x 60 | 0.25 | 0.281 ± 0.0014 | 1/6 | 149 | 6 | 359 |
| IEPY 20 (20dpi) | 30 x 30 | 60 x 60 | 0.25 | 0.421 ± 0.0052 | 1/6 | 227 | 7 | 616 |
| EEcont 6 (20dpi) | 30 x 30 | 60 x 60 | 0.25 | 0.408 ± 0.0099 | 1/6 | 238 | 6 | 693 |
| EEcont 7 (20dpi) | 30 x 30 | 60 x 60 | 0.25 | 0.316 ± 0.0017 | 1/6 | 182 | 5 | 479 |
| EEcont 23 (20dpi) | 30 x 30 | 60 x 60 | 0.25 | 0.337 ± 0.0025 | 1/6 | 170 | 5 | 432 |
| EEcont 25 (20dpi) | 30 x 30 | 60 x 60 | 0.25 | 0.318 ± 0.0016 | 1/6 | 167 | 5 | 447 |
| EEPY 10 (20dpi) | 30 x 30 | 60 x 60 | 0.25 | 0.420 ± 0.0059 | 1/6 | 241 | 7 | 667 |
| EEPY 18 (20dpi) | 30 x 30 | 60 x 60 | 0.25 | 0.422 ± 0.0090 | 1/6 | 208 | 7 | 544 |
| EEPY 21 (20dpi) | 30 x 30 | 60 x 60 | 0.25 | 0.334 ± 0.0010 | 1/6 | 149 | 5 | 404 |
| EEPY 22 (20dpi) | 30 x 30 | 60 x 60 | 0.25 | 0.423 ± 0.0171 | 1/6 | 226 | 7 | 706 |

^(2)^ All evaluations were performed with a 60X objective lens (N.A. 1.4; D.F. 0.75 µm).
